# Cellular Phenotype-Dependent and -Independent Effects of Vitamin C on the Renewal and Gene Expression of Mouse Embryonic Fibroblasts

**DOI:** 10.1371/journal.pone.0032957

**Published:** 2012-03-13

**Authors:** Shiu-Ming Kuo, Lana R. Burl, Zihua Hu

**Affiliations:** 1 Department of Exercise and Nutrition Sciences, University at Buffalo, Buffalo, New York, United States of America; 2 Department of Biochemistry, University at Buffalo, Buffalo, New York, United States of America; 3 Center for Computational Research, University at Buffalo, Buffalo, New York, United States of America; 4 Department of Biostatistics, University at Buffalo, Buffalo, New York, United States of America; Paris Institute of Technology for Life, Food and Environmental Sciences, France

## Abstract

Vitamin C has been shown to delay the cellular senescence and was considered a candidate for chemoprevention and cancer therapy. To understand the reported contrasting roles of vitamin C: growth-promoting in the primary cells and growth-inhibiting in cancer cells, primary mouse embryonic fibroblasts (MEF) and their isogenic spontaneously immortalized fibroblasts with unlimited cell division potential were used as the model pair. We used microarray gene expression profiling to show that the immortalized MEF possess human cancer gene expression fingerprints including a pattern of up-regulation of inflammatory response-related genes. Using the MEF model, we found that a physiological treatment level of vitamin C (10^−5^ M), but not other unrelated antioxidants, enhanced cell growth. The growth-promoting effect was associated with a pattern of enhanced expression of cell cycle- and cell division-related genes in both primary and immortalized cells. In the immortalized MEF, physiological treatment levels of vitamin C also enhanced the expression of immortalization-associated genes including a down-regulation of genes in the extracellular matrix functional category. In contrast, confocal immunofluorescence imaging of the primary MEF suggested an increase in collagen IV protein upon vitamin C treatment. Similar to the cancer cells, the growth-inhibitory effect of the redox-active form of vitamin C was preferentially observed in immortalized MEF. All effects of vitamin C required its intracellular presence since the transporter-deficient SVCT2−/− MEF did not respond to vitamin C. SVCT2−/− MEF divided and became immortalized readily indicating little dependence on vitamin C for the cell division. Immortalized SVCT2−/− MEF required higher concentration of vitamin C for the growth inhibition compared to the immortalized wildtype MEF suggesting an intracellular vitamin C toxicity. The relevance of our observation in aging and human cancer prevention was discussed.

## Introduction

Vitamin C was first characterized as a coenzyme and a water-soluble antioxidant. Additional biological activities of vitamin C have been described subsequently. In primary cell culture, its ability to promote growth was reported to be due to an increased cell division and cell differentiation [1]–[4]. A related property of vitamin C to delay senescence [5] and enhance primary fibroblast reprogramming to pluripotency was further demonstrated [6]. These in vitro observations made at physiologically relevant concentrations of vitamin C (10^−5^ M) are consistent with the results of in vivo studies. Patients with premature aging disorder, Werner Syndrome, display accelerated senescence in their fibroblasts [7] and develop rare forms of cancer [8]. In a mouse model of Werner Syndrome, vitamin C was found to promote normal growth and delay senescence [9]. In this paper, gene expression profiling and immunofluorescence imaging were conducted to facilitate the understanding of molecular mechanisms mediating vitamin C-dependent growth promotion.

Little is known about the effect of vitamin C, at physiologically relevant conditions, on the growth of immortalized cells. Treating cancer cells with pharmacological levels of vitamin C (10^−4^ M and above) is known to lead to cell cycle arrest and apoptosis [10]–[12], as well as gene expression changes [13]. While these observations were used to support the chemotherapeutic potential of vitamin C, a recent meta-analysis failed to conclude on the efficacy of vitamin C [14]. Mutant mice that could not synthesize vitamin C endogenously, a trait similar to humans [15], had more aggressive growth of implanted tumor after vitamin C supplementation [16]. In vitro studies using pharmacological doses of vitamin C were further complicated by the known free radical-generating interaction between vitamin C and culture medium components [17]. In this paper, vitamin C dose-dependent effects were analyzed in immortalized cells using redox-active and -inactive vitamin C. Gene expression profiles of primary and immortalized cells were also compared after the same vitamin C treatment.

Mouse embryonic fibroblasts (MEF) were used as the cell model in this study. The vitamin C-synthesizing ability of mouse is limited to hepatocytes [18] so MEF mimic human cells in their exogenous vitamin C requirement. In addition, MEF can undergo spontaneous immortalization, thus allowing the comparison of a single cell type at two phenotypes: primary MEF with limited mitotic life span; and immortalized MEF. Immortalization is considered a link between normal and tumorigenic cells [19], [20]. The relevancy of immortalized MEF to certain human cancer was previously discussed [21], [22]. Fibroblasts also contribute to the cancer stem cell niche [23] and thus the response of fibroblasts to vitamin C could have additional implications. Besides the wildtype MEF, MEF from embryos that do not express functional sodium-dependent vitamin C transporter 2 (SVCT2−/−) were also used in the study to distinguish the effects of intracellular and extracellular vitamin C.

## Materials and Methods

### Materials

L-[carboxyl-^14^C]ascorbic acid (13 mCi/mmol) was purchased from Amersham Biosciences (Buckingshire, England) and [methyl-^3^H]thymidine was from ICN Pharmaceuticals, Inc. (Irvine, CA). L-ascorbic acid (SigmaUltra) and L-ascorbic acid 2-phosphate (sesquimagnesium salt) were from Sigma Aldrich (St. Louis, MO). D-(-)-isoascorbic acid (>99% pure) was from Fluka. All cell culture reagents were from Invitrogen Corp. (Carlsbad, CA) except characterized fetal bovine serum, which was purchased from Hyclone, USA (Logan, UT). EcoLite™(+) was used as the scintillation fluid (ICN Pharmaceuticals, Inc.). All other chemicals used were analytical or molecular biology grade.

### Mouse embryonic fibroblast generation, propagation and treatment

All MEF were collected from a transgenic mouse colony maintained in University at Buffalo specific pathogen-free facility [24] following protocols approved by the Institutional Animal Care and Use Committee. Embryos at ED 13.5 were used and genotyping by PCR was described before [24]. Standard high-glucose DMEM medium supplemented with penicillin, streptomycin and 10% FBS was used for the culturing [25]. To enrich fibroblasts, cells that were not attached to the flask surface after 1-hour culturing were removed. MEF were immortalized by the original 3T3 method [19] or a published limited medium method [20]. Immortalization is characterized by an increase in the rate of growth after a prolonged period of growth arrest and is associated with the death of most cells and emergence of small numbers of colonies [19]. We were able to consistently establish immortalized MEF cell lines (N = 18) and propagate them for more than 40 passages. Most experiments were repeated using MEF from different embryos. Cell treatment was always started the day after seeding, and carried out daily by adding stock solutions (dissolved in phosphate buffered saline) to the growth medium. There was no medium change between cell seeding and harvesting during cell propagation experiment. Some experiments, when indicated, were performed with MEFs propagated in T-25 flask from one passage to the next consistently in the presence of 20 µM ascorbate 2-phosphate. To determine the effect of vitamin C or indicated chemicals on the cell growth, cells propagated with or without vitamin C were harvested from their T-25 flasks and grown in 12-well plates for treatment comparison. The total amount of protein in a monolayer was used to monitor cell growth. A modified Lowry assay [26] with bovine serum albumin as the standard was used.

### ^3^H-thymidine incorporation

A modified ^3^H-thymidine incorporation [27] procedure was used to determine cell division. Briefly, to perform the ^3^H-thymidine incorporation experiment, ^3^H-thymidine (1 µCi per well of 6-well plate) was added to the cell monolayer after a medium change. After the 4-hour incorporation, the labeled DNA in trichloroacetic acid-lysed cells was collected and the radioactivity determined by the liquid scintillation counting using Beckman coulter LS6500 Scintillation Counter. The incorporation was specific as the negative control samples (addition of ^3^H-thymidine without incubation) had only background radioactivity.

### Vitamin C accumulation

The ability of MEF to take up ascorbic acid through sodium-dependent and -independent pathways was determined by ^14^C-ascorbate transport assay as previously described [28]. Briefly, cell monolayers grown in 6-well culture plates were rinsed and incubated with Hanks' balanced salt solution in a 37°C incubator. Hanks' balanced salt solution containing N-methyl-glucamine substituting sodium was used to determine sodium-independent transport. ^14^C-ascorbate (10 µM) was added and the uptake was terminated after 15 minute incubation by the removal of ^14^C-ascorbate-containing Hanks' balanced salt solution. Monolayers were then rinsed three times with ice-cold phosphate buffer saline before lysed by 0.5% SDS for the radioactivity and protein measurements. Triplicate wells of cells were used for the transport assay and the results represented the mean±SD of three independent wells after normalization by total protein in each well. Experiments were repeated with different passages of wildtype and SVCT2−/− MEF and the results were similar.

### Statistical analyses of cell samples

Statview and Super-ANOVA v.1.11 (Abacus Concepts Inc., Berkeley, CA) were used to analyze the measurement in total protein, thymidine incorporation and vitamin C uptake. The Student's t-test was used to compare between two groups and one-way ANOVA was performed along with Bonferroni/Dunn test for the multiple comparison. Effects with p<0.05 were considered significant.

### Confocal imaging

Primary embryonic fibroblasts were grown on the glass coverslips with 4-day treatment of phosphate-buffered saline (vehicle) or 20 µM vitamin C. Cells were fixed with 4% paraformaldehyde and then treated with 10% DMSO before the antibody incubation. To visualize collagen IV, primary rabbit anti-collagen IV antibody (sc-11360, Santa Cruz Biotechnology, Inc.) was used in conjunction with Alexa Fluor® 488 goat anti-rabbit IgG (H+L) (A11008, Invitrogen Corp). To visualize F-actin distribution, Alexa Fluor® 568 phalloidin (A12380, Invitrogen Corp.) was used. The manufacturer's protocols (Invitrogen Corp. Carlsbad, CA) were followed for the staining. A. Zeiss LSM510 confocal microscope with 63× objective lens (UB Confocal and 3-D imaging facility) was used to capture images and the same confocal setting was used for the imaging of control and vitamin C-treated MEF. Within each genotype, the control and treated cells were from the same passage and processed at the same day for imaging. Multiple images were acquired from triplicate cell samples of the same genotype/treatment. Representative confocal images were processed with Volocity 4.0.1 software (Perkin Elmer, Inc.) to generate 3-D volumes and X-Z sections.

### RNA isolation and microarray hybridization

P4 (representing the primary cells) and P32 (representing the immortalized cells) of MEF were used for gene expression profiling. There were four treatment groups: primary cells with or without 2-day 20 µM ascorbate 2-phosphate treatment, and immortalized cells with or without 2-day 20 µM ascorbate 2-phosphate treatment. Total RNA was extracted from triplicate cell monolayers using MasterPure™ RNA Purification Kit (Epicentre, Madison, WI) following the manufacturer's protocol. The quality of RNA was determined by the OD260/OD280 ratio (greater than 1.9) and verified using Agilent 2100 bioanalyzer (Agilent Technologies, Santa Clara , CA). The concentration of RNA was determined by OD260. Whole mouse genome 4×44 K gene expression single-color microarray kit (US00044437) [29] from the same batch (Agilent Technologies, Santa Clara , CA), verified using NCBI genome Build 32, was used for gene expression analysis following the manufacturer's protocol by Empire Genomics, Buffalo, NY (an Agilent-certified facility). The Agilent array used 60mer oligonucleotide probes including positive and negative controls as well as redundant detection of some transcripts at different areas of the array using the same or different probes. All twelve samples in the study were processed on the same day. They were randomly assigned to three slides for hybridization.

### Microarray Data analysis

Data analysis was performed on twelve arrays for four treatment groups (N = 3 per group, RNA samples from three independent wells in each treatment group). The Lowess method was first used for intensity-dependent normalization, and then followed by global normalization of the probe expression level. The goal was to adjust the median expression values of all arrays to the same scale. One experimental array (immortalized MEF without vitamin C treatment) was randomly selected as the baseline array and its median value (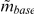) was used for the normalization of remaining arrays: 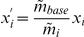.

Only probes with expression levels above the mean of negative controls in at least 50% of the samples were used for the statistical comparison. The results of normalization and MA plots are included as Figure S1. The regularized *t*-test [30], which is good for limited numbers of replicates, was employed to detect significantly treatment effects. For multiple test correction, the false discovery rate (*q*-value<0.1) was controlled by adjusting the *p*-values using the following formula:
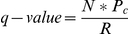
where *N* is the number of genes in the test, and *R* is the ascending rank order of the respective *p*-value at certain cutoff *P_c_*. A statistically valid list of differentially expressed genes was then sorted based on folds of treatment effect and grouped as shown in Tables 1–3. To objectively determine the effect of treatment on functionally cooperative genes, each group of DEGs was analyzed by the tools in Database for Annotation, Visualization and Integrated Discovery (DAVID; http://david.abcc.ncifcrf.gov/) version 6.7 [31], [32]. This functional annotation is to determine statistically significant over-representation of functional categories due to each treatment. A gene could belong to multiple functional categories.

**Table 1:** Summary of the effect of vitamin C treatment on the gene expression of primary mouse embryonic fibroblasts as determined by gene expression microarray analysis^a^.

Degree of effect	Number of probes affected^b^	Gene expression profiling for functional enrichment^c^
p<0.01		
↑ expression	1575	
↓ expression	225	
p<0.01, >1.5-fold effect		
↑ expression	219 (205)	Cell cycle (13) p = 9.1E-3
		Cell division (10) p = 3.4E-3
↓ expression	109 (108)	
p<0.01, >3-fold effect		
↑ expression	0	
↓ expression	4 (4)	

a The passage 4 primary embryonic fibroblasts were treated with 20 µM ascorbate 2-phosphate for 2 days before RNA harvesting. The gene expression level in the vitamin C-treated primary MEF was divided by the level in the untreated primary MEF to obtain the fold change.

b Vitamin C treatment significantly affected the expression of these probes/genes. Multiple probes may be used to quantify the expression of the same gene. The number in the parenthesis represents the number of genes affected.

c p value shown is Benjamini.

**Table 2 pone-0032957-t002:** Summary of the effect of immortalization on the gene expression of mouse embryonic fibroblasts as determined by gene expression microarray analysis^a^.

Degree of effect	Number of probes affected^b^	Gene expression profiling for functional enrichment^c^
p<0.01		
↑ expression	4810	
↓ expression	4210	
p<0.01, >1.5-fold effect		
↑ expression	3968	
↓ expression	3343	
p<0.01, >3-fold effect		
↑ expression	1147 (945)	Glycoprotein (218)
		Inflammatory response (30)
↓ expression	1319 (1011)	Glycoprotein (284)
		Extracellular matrix (55)
		Tissue morphogenesis (37)

a The expression level of genes in the untreated immortalizaed (passage 32) MEF was divided by levels in the untreated primary (passage 4) MEF to obtain fold changes.

b Immortalization significantly affected the expression of these probes/genes. Multiple probes may be used to quantify the expression of the same gene. The number in the parenthesis represents the number of genes affected.

c p value shown is Benjamini.

**Table 3:** Summary of the effect of vitamin C treatment on the gene expression of immortalized mouse embryonic fibroblasts as determined by gene expression microarray analysis^a^.

Degree of effect	Number of probes affected^b^	Gene expression profiling for functional enrichment^c^
p<0.01		
↑ expression	1844	
↓ expression	1276	
p<0.01, >1.5-fold effect		
↑ expression	1284 (1048)	Cell cycle (99) p = 3.0E-26
		Cell division (59) p = 1.0E-23
		
↓ expression	1060 (840)	Extracell matrix(63)p = 1.5E-26
		Cell adhesion (57) p = 2.4E-10
		Collagen (21) p = 2.9E-10
p<0.01, >3-fold effect		
↑ expression	97 (88)	
↓ expression	276 (229)	Extracell matrix(31)p = 3.5E-19

a Immortalized embryonic fibroblasts (p32) were treated with 20 µM ascorbate 2-phosphate for 2 days before RNA harvesting. The gene expression level in the vitamin C-treated immortalized wildtype MEF was divided by the level in the untreated immortalized wildtype MEF to obtain the fold change.

b Vitamin C treatment significantly affected the expression of these probes/genes. Multiple probes may be used to quantify the expression of the same gene. The number in the parenthesis represents the number of genes affected.

c p value shown is Benjamini.

### qPCR validation of microarray analysis

cDNA was generated from total RNA by reverse transcription using oligo-dT 15 primer (Promega, Madison, WI) and Omniscript reverse transcriptase (Qiagen, Valencia, CA). Primers used for qPCR were Adamts4: forward, AGCAAGCAGTCGGGCTCCTT, reverse, GCGTAAGAACCGTCAGAAAG; CNN1: forward, TGCGAATTTATCAACAAGCTG C, reverse, CCCCATACTTGGTAATGGCTTTG; Csf1: forward, 5GAGCAGGAGTATTGCCAAG, reverse GCCTTCTTTAGGTAGCAAACAG; Igf2: forward, GTGCTGCATCGCTGCTTAC, reverse, ACGTCCCTCTCGGACTTGG; MmP13: forward, GTGATGATGATGATGATGACCTG, reverse, GCATTTCTCGGAGCCTGTC; Ptprdf1: forward, CTGCTCTCGTGATGCTTGGTT, reverse, ATCCACGTAATTCGAGGCTTG.

Quantitative PCR reaction was performed in MyiQ iCycler (UB Confocal and 3-D imaging facility) using SsoAdvanced SYBR Green Supermix (Bio-Rad Labs, Richmond, CA) and the expression of GAPDH was used for normalization (forward, ATCCCATCACCATCTTCCAG, reverse, GCCATCACGCCACAGTTTCC). Similar to the microarray analysis, triplicates (three independent wells of cells) were used for the qPCR analyses. The correlation between the fold changes from microarray analysis and from qPCR is shown in the Figure S2 (r = 0.997).

## Results

### Physiological level vitamin C, but not other antioxidants, promotes the growth of primary MEF

**Figure 1:**
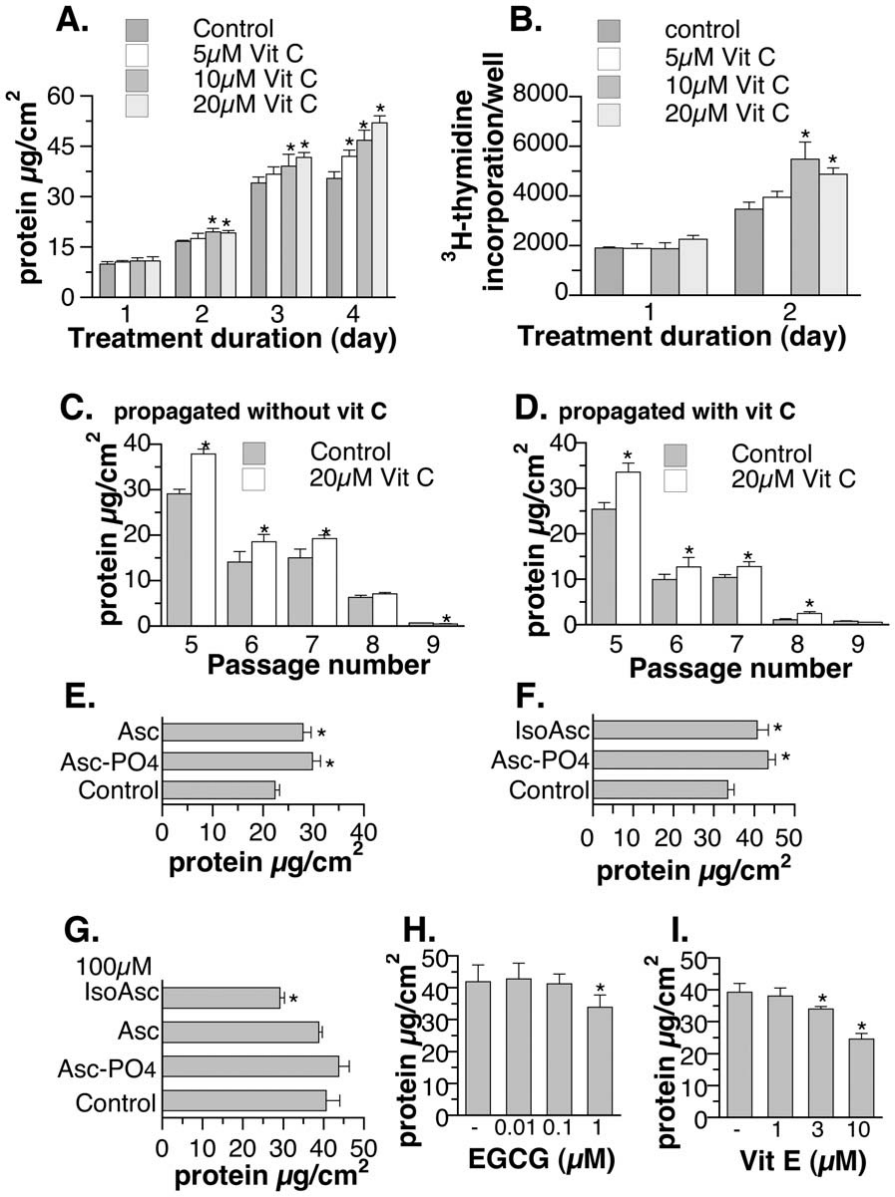
Dose- and treatment duration-dependent effect of vitamin C and its analogs on primary mouse embryonic fibroblasts. Cells propagated in normal medium at low passages (before passage 6 except 1E and 1F) were used for the study. They were seeded in cell culture wells at 2×10^4^ cells/cm^2^ and treated every day starting the day after seeding. They were harvested 24 hours after the last treatment. There was no medium change from cell seeding to the end of ascorbate 2-phopshate treatment. The medium was changed before the start of the thymidine incorporation experiment as a part of the experimental procedure. (A) Effect of 1–4 days of treatment with 5–20 µM ascorbate 2-phosphate on total protein; (B) Effect of 1–2 days of treatment with 5–20 µM ascorbate 2-phosphate on thymidine incorporation; (C)(D) Passage-dependent effect of ascorbate 2-phosphate on mouse embryonic fibroblasts propogated in the absence (C) or presence (D) of 20 µM ascorbate 2-phosphate; (E) (F) Effect of 4-day treatment of 20 µM ascorbic acid (E) or 20 µM isoascorbate (F) on total protein; (G–I) Effect of 4-day treatment of (G) 100 µM vitamin C or analog, (H) 0.01–1 µM (-)-epigallocatechin-3-gallate (EGCG), or (I) 1–10 µM alpha-tocopherol succinate (Vit E) on total protein. All data shown represent means±S.D. of 3–4 independent wells. *significantly different from the control cells without treatment at p<0.05.

The effect of vitamin C on the growth of primary MEF is shown in Fig. 1. To prevent pro-oxidant activity due to the interaction of ascorbic acid with medium components [17], a previously characterized pro-drug form of vitamin C [1], ascorbate 2-phosphate, was used for the supplement throughout the study unless indicated otherwise. As shown in Fig. 1A, dose-dependent effects were observed, and 10 and 20 µM supplementation led to a consistent increase in total cellular protein starting two days after the initiation of treatment. The concentration range was chosen because 10 µM is the minimal plasma concentration required for the prevention of clinical vitamin C deficiency. As shown in Fig. 1B, two-day treatment with 10 or 20 µM vitamin C significantly increased thymidine incorporation. Thus, increasing cell division contributed to the growth promotion by vitamin C. The growth-promotion by vitamin C can be observed throughout the serial passaging of primary MEF (Fig. 1C). Briefly, MEF propagated in T-25 flasks were harvested at each indicated passage to seed into 12-well plates for the experiment. The MEF were treated with vehicle or 20 µM ascorbate 2-phosphate. In addition, MEF were also propagated in T-25 flasks with 20 µM ascorbate 2-phosphate in the medium (Fig. 1D). At each indicated passage, cells were harvested from the T-25 flask and seeded into 12-well plates for the experiment. Similar to the cells in Fig. 1C, cells in 12-well plates were treated with vehicle or 20 µM ascorbate 2-phosphate. Similar to the cells in Fig. 1C, 20 µM ascorbate 2-phosphate treatment increased cell growth at every passage (Fig. 1D).

As shown in Fig. 1E and 1F, 20 µM ascorbic acid or 20 µM isoascorbic acid also significantly increased the total cellular protein after four days of treatment. At higher concentration (100 µM), the growth-promoting effect disappeared for ascorbate 2-phosphate and ascorbic acid, and isoascorbate slightly inhibited the growth (Fig. 1G). Although vitamin C is known as an antioxidant, primary MEF treated with other structurally unrelated antioxidants, (-)-epigallocatechin-3-gallate (0.01–1 µM) (Fig. 1H) and vitamin E (1–10 µM) [33], did not show any increase in total cellular protein. Control MEF and MEF treated with 20 µM ascorbate 2-phosphate for four days showed no differences in the level of malondialdehyde, a lipid-oxidation byproduct (results not shown).

### Confocal immunofluorescene imaging analysis of vitamin C-treated MEF

**Figure 2:**
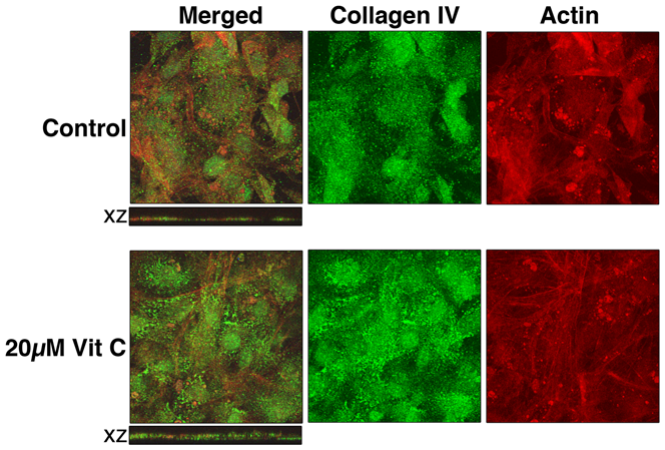
Confocal immunofluorescence visualization of collagen IV and ß-actin expression in primary embryonic fibroblasts with (bottom row) and without (top row) 4-day ascorbate 2-phosphate treatment. There was no medium change between cell seeding and harvesting for staining. Composite confocal images of cells stained for collagen IV (second column) and F-actin (third column) were merged (first column) and images of X-Z sections were captured. The size of the merged X-Y image was 140 µm×140 µm. The X-Z section was taken from a stack of thirty-five 0.46 µm slices.

One major function of vitamin C is to assist the post-translational modification of collagen. Collagen IV is mainly found in the basement membrane [34] where fibroblasts reside and function [35]. To determine the effect of vitamin C on intracellular collagen IV protein level, primary MEF (p4) were grown in the absence (Fig. 2, top row) or presence (Fig. 2, bottom row) of 20 µM vitamin C, over four days of treatment. Compared to the untreated MEF of the same passage, intracellular collagen appeared more prominent with the vitamin C treatment especially in the X-Z section (Fig. 2 compared the top and bottom rows). In contrast, F-actin, a component of cytoskeleton, did not appear to increase in vitamin C-treated cells (Fig. 2). MEF also gained cell height upon vitamin C treatment (Fig. 2, X-Z sections), which can contribute to the increase in total protein observed in Fig. 1A and C–F.

### Using vitamin C transporter-null MEF to confirm the role of intracellular vitamin C in the growth of primary MEF

**Figure 3:**
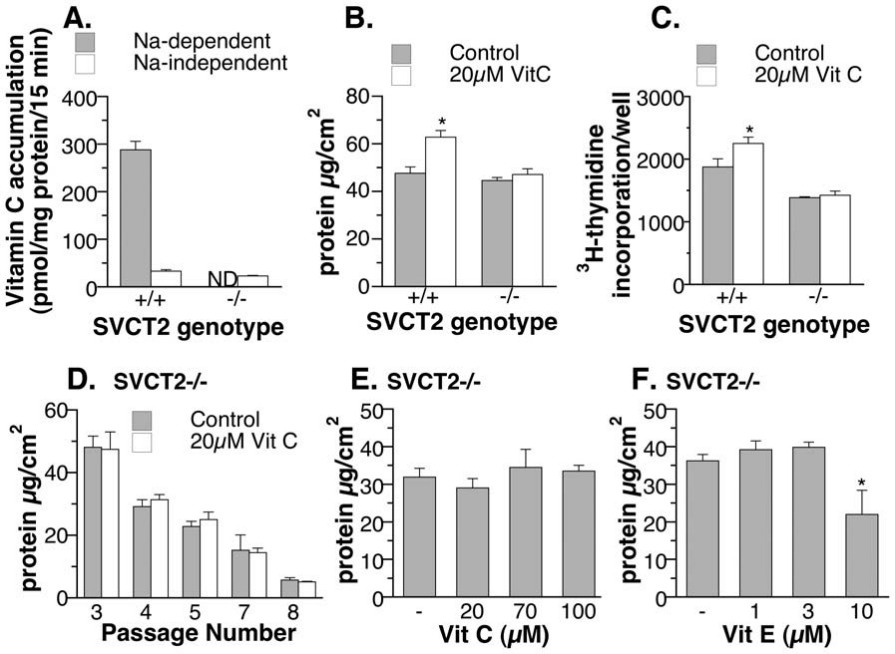
Vitamin C in the medium does not affect SVCT2−/− embryonic fibroblasts. (A) SVCT2−/− fibroblasts do not have sodium-dependent vitamin C transport activity. ND: determined but not detected. (B,C) SVCT2−/− fibroblasts do not show vitamin C treatment-dependent increase in (B) total protein (measured after 4-day treatment with 20 µM ascorbate 2-phosphate) or (C) thymidine incorporation (measured after 2-day treatment with 20 µM ascorbate 2-phosphate). There was no medium change from cell seeding to the end of ascorbate 2-phopshate treatment. The medium was changed before the start of the thymidine incorporation experiment as a part of the experimental procedure. Unlike the wildtype fibroblasts, SVCT2−/− fibroblasts did not show (D) passage-dependent; or (E) dose-dependent response to ascorbate 2-phosphate in the medium; but did display (F) dose-dependent response to vitamin E in the medium. All data shown represent means±S.D. of 3–4 independent wells. *significantly different from control cells without treatment at p<0.05.

**Figure 4:**
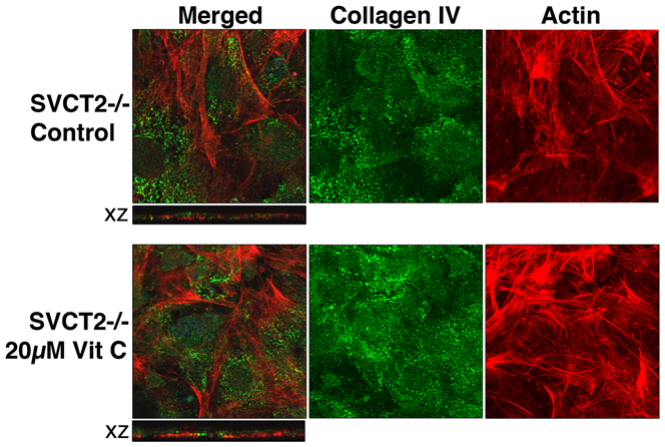
Confocal immunofluorescence visualization of collagen IV and ß-actin expression in primary SVCT2−/− embryonic fibroblasts with (bottom row) and without (top row) 4-day ascorbate 2-phosphate treatment. There was no medium change between the cell seeding and harvesting for staining. Composite confocal images of cells stained for collagen IV (second column) and F-actin (third column) were merged (first column) and images of X-Z section were captured. The size of the merged X-Y image was 140 µm×140 µm. The X-Z section was from a stack of thirty-five 0.46 µm slices.

To demonstrate the importance of intracellular vitamin C for the effects shown in Figs. 1 and 2, sodium-dependent vitamin C transporter (SVCT2)-null MEF was used for comparison (Fig. 3). Vitamin C is made in hepatocytes of vitamin C-synthesizing species such as mice and its distribution to extrahepatic tissues mostly depends on cellular SVCT2 [36]. SVCT1 is not known to be expressed in the fibroblasts [37] and we did not detect the presence of SVCT1 in our MEF either (results not shown). SVCT2−/− MEF did not have sodium-dependent vitamin C transport activity (Fig. 3A) but did show limited sodium-independent vitamin C transport activity similar to the SVCT2+/+ MEF (Fig. 3A). SVCT2-null embryonic fibroblasts grow well indicating that the need of vitamin C for cell division is minimal. Different from SVCT2+/+ MEF, SVCT2−/− MEF showed no increase in the total protein (Fig. 3B); nor in the thymidine incorporation after 20 µM vitamin C treatment (Fig. 3C). This lack of response to vitamin C treatment persisted throughout the serial passaging of SVCT2−/− MEF (Fig. 3D) and at 20, 70 and 100 µM of ascorbate 2-phosphate treatment (Fig. 3E). In contrast, the growth inhibitory effect of 100 µM vitamin E observed in wildtype MEF (Fig. 1I) was similarly found in SVCT2−/− MEF (Fig. 3F). In confocal immunofluorescence study, treatment of SVCT2−/− MEF with 20 µM vitamin C for 4 days also did not increase the cell height (Fig. 4, X-Z sections), which is consistent with the lack of increase in total protein after vitamin C treatment (Fig. 3B and D, E). Based on the results in Figs. 3 and 4, intracellular vitamin C accumulation is needed to realize the growth-promoting effect of vitamin C.

### Vitamin C and the gene expression in wildtype primary MEF

Microarray analysis was performed to determine whether two days of vitamin C treatment affected the expression of cell growth-related genes. We chose the duration because two days of treatment with 20 µM vitamin C was sufficient to increase total protein and thymidine incorporation significantly (Fig. 1A, B). The treatment did lead to changes in gene expression (Table 1). Of the genes that showed significant overexpression at greater than 1.5 fold, we performed functional annotation using DAVID and found enrichment in cell cycle (13 genes) and cell division (10 genes) functional categories. A complete list of these genes is included in the Table S1. Of these, four genes, Asp (abnormal spindle)-like, cell division cycle 25C, cyclin B1, and RAD15 homolog, were previously found to increase in expression (2.5–2.9 folds) when primary human skin fibroblasts were treated with 100 µM of ascorbate 2-phosphate for five days [38]. Results from the gene expression analysis is consistent with the thymidine incorporation finding in Fig. 1B that vitamin C treatment of the primary MEF increased cell division.

### MEF immortalization and gene expression profiling of the immortalized MEF

**Figure 5:**
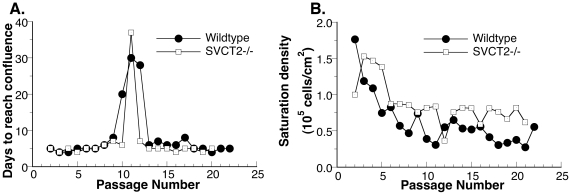
The immortalization of wildtype and SVCT2−/− fibroblasts. Wildtype and SVCT2−/− mouse embryonic fibroblasts were carried to beyond immortalization following the published limited medium method [20]. The immortalization process was associated with (A) a significant increase in days to reach confluence around passage 11. Once immortalized, MEF regained rapid cell division. (B) Saturation density was higher in both wildtype and SVCT2−/− primary fibroblasts compared to their respective immortalized fibroblasts.

Wildtype and SVCT2−/− MEF reached immortalization similarly (Fig. 5A and B). This observation again indicated a lack of significant need of vitamin C by the cell division machinery. Immortalization was associated with significant gene expression changes (Table 2). Functional annotation revealed that among the genes that over-expressed greater than 3-fold, there was enrichment in genes that represent glycoproteins (218 genes) and genes involved in the inflammatory response (30 genes). A complete list of these up-regulated genes is included in the supplemental material (Table S2). Of the genes showed more than a 3-fold decrease in expression in immortalized MEF compared to the primary MEF (Table 2), enrichment of glycoproteins (284 genes) was also observed. In addition, down-regulation of genes related to the extracellular matrix (55 genes) was also significant. A complete list of the down-regulated genes is included in the supplemental material (Table S3).

### Effect of vitamin C on the growth of immortalized MEF

**Figure 6:**
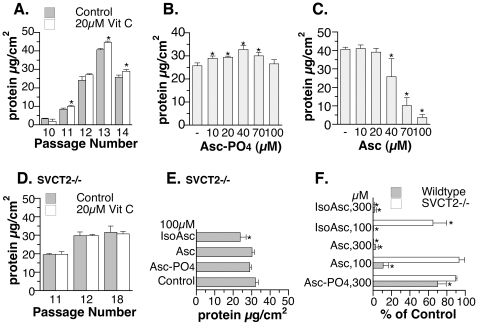
The effect of vitamin C treatment on immortalized wildtype and SVCT2−/− fibroblasts. (A) The effect of 4-day 20 µM ascorbate 2-phosphate treatment on the total protein of immortalized wildtype fibroblasts. (B,C) Dose-dependent effect of 4-day treatment with (B) ascorbte 2-phosphate and (C) ascorbate on the immortalized wildtype fibroblasts. (D) Immortalized SVCT2−/− MEF did not respond to 4-day 20 µM ascorbate 2-phosphate treatment. (E) Immortalized SVCT2−/− fibroblasts were inhibited only by 4-day 100 µM isoascorbate treatment. (F) Immortalized wildtype fibroblasts were more sensitive to the inhibitory effect of pharmacological concentrations of vitamin C and analog compared to immortalized SVCT2−/− fibroblasts (4-day treatment). All data shown represent means±S.D. of 3–4 independent wells. Some values and error bars are too small to be clearly visible on the graph. *significantly different from the control cells without treatment at p<0.05.

The immortalized MEF grew slowly initially but small growth-promoting effect of vitamin C was consistently observed starting passage 11 (Fig. 6A). This vitamin C treatment-induced increase in total protein was less than those observed in primary MEF (Fig. 6A vs. 1C, 1D). The growth-promoting effect of vitamin C was also dose-dependent (Fig. 6B & 6C). The growth-inhibitory effect observed at higher free ascorbate concentrations (Fig. 6C) is consistent with the reported cytotoxic pro-oxidant effect of ascorbate in cancer cells [11], [39], [40]. The biphasic effect of vitamin C was also observed in an unrelated line of human colon cancer cells, Caco2. Ascorbate 2-phosphate at 40 µM showed growth-promoting effect while ascorbate, the redox active form, at 40 µM or higher inhibited cell growth (results not shown). Immortalized SVCT2−/− MEF, as predicted from its lack of ability to acquire vitamin C from medium (Fig. 3A), showed no response to physiological treatment levels of vitamin C (Fig. 6D). Also, different from the wildtype MEF, immortalized SVCT2−/− MEF was not sensitive to the inhibition by 100 µM free ascorbate and was only slightly inhibited by 100 µM isoascorbate in the medium (comparing Fig. 6C to 6E, also 6F). As shown in Fig. 6F, 300 µM ascorbate and isoascorbate were needed to reach the same degree of inhibition in immortalized SVCT2−/− MEF (open bar). The inhibition at 300 µM could be a result of a free radical-generating interaction between ascorbate and isoascorbate with medium components [11], [39], [40] because 300 µM non-redox ascorbate 2-phosphate had minimal effect on immortalized SVCT2−/− MEF (Fig. 6F). At 1 mM, ascorbate and ascorbate 2-phosphate treatments led to more inhibition of cell growth in both cell types which is consistent with the widely reported dose-dependent growth inhibition by vitamin C at 10^−4^ to 10^−3^ M (for example, [13]).

### Effect of vitamin C on the gene expression of immortalized MEF

Treatment of immortalized wildtype MEF with physiological levels of vitamin C was also associated with gene expression changes (Table 3). A summarized comparison between the responses of primary and immortalized MEF to the treatment of vitamin C is included in Figure S3. For genes that showed at least 1.5 fold significant increase in expression, functional annotation revealed that there was enrichment in genes involved in cell cycle (99 genes) and cell division (59 genes). A complete list of these up-regulated genes is included in the supplemental material (Table S4). This enrichment shared a similarity with the gene expression pattern of primary MEF after vitamin C treatment (Table 1). Interestingly, up-regulation was observed for Asp (abnormal spindle)-like, cell division cycle 25C, cyclin B1, and RAD15 homolog, the same four genes that showed increases after vitamin C treatment of primary human skin fibroblasts [38] and primary MEF (Table 1). For the genes that showed at least 1.5-fold significant decrease in expression in response to 20 µM vitamin C treatment, there was enrichment in genes relating to extracellular matrix (63 genes), cell adhesion (57 genes) and collagen (21 genes). A complete list of genes that were significantly down-regulated by vitamin C in immortalized MEF is included in the supplemental material (Table S5). There were 31 genes in the extracellular matrix functional category that showed at least 3-fold down-regulation in immortalized MEF after physiological levels of vitamin C treatment (last row of Table 3). A list of the genes is also included in the supplemental material (Table S5). When the results in Table 3 were compared to the results in Table 2, we found that the immortalization process and vitamin C treatment of immortalized MEF were similarly associated with a decreased expression of extracellular matrix genes. To examine if vitamin C treatment of immortalized MEF led to further up-regulation of genes that were increased by the immortalization, 20 genes that showed the greatest over-expression upon immortalization were compiled (Table 4). Most of these genes or their family members were also found to be over-expressed in human cancers [41]–[56]. These genes had a mean signal level of 123±289 as primary MEF (ranging from 8-1173), which was higher than the signal level of the negative control in the array (around 5). As shown in the last column of Table 4, vitamin C treatment led to further increase in the expression of some genes but not all. Two genes, Cyp2f2 and Sp100, that increased expression upon immortalization, showed less expression in vitamin C-treated immortalized MEF. Although genes involved in protein synthesis were found to be affected by 300–800 µM vitamin C treatment [13], under our physiological treatment condition (20 µM ascorbate 2-phosphate), genes involved in protein systhesis were not affected in primary or immortalized MEF. This observed difference is not surprising as growth inhibition was observed by Belin et al at high concentrations [13] whereas our treatment showed growth stimulation (Fig. 1 and 6). In summary, vitamin C treatment of immortalized MEF led to changes that were in the same direction as the changes observed in the immortalization process.

**Table 4:** Effect of vitamin C treatment on the 20 genes that had maximal increase in expression upon immortalization in mouse embryonic fibroblasts (MEF).

		Fold of changes in expression^a^	
Gene	Gene description	Immortalization^b^	vitamin C^c^
Plf2	proliferin 2	5985 (p = 1.5E-06)	2.85 (p = 0.002)
Mrpplf4	mitogen regulated protein, proliferin 4	2863 (p = 1.9E-06)	2.37 (p = 0.004)
**Car8** d	carbonic anhydrase 8	67 (p = 7.7E-06)	1.65 (p = 0.004)
**Trim30**	tripartite motif protein 30	66 (p = 3.9E-05)	-
**Ccl6**	chemokine (C-C motif) ligand 6	64 (p = 0.0006)	1.84 (p = 0.002)
**Naalad2**	N-acetylated alpha-linked acidic dipeptidase 2	58 (p = 2.9E-05)	-
**Cst6**	cystatin E/M	57 (p = 3.7E-07)	-
**Cyp2f2**	cytochrome P450, family 2-f, polypeptide 2	56 (p = 5.5E-05)	−2.37 (p = 0.007)
**Sp100**	nuclear antigen Sp100	53 (p = 2.5E-05)	−1.71 (p = 0.0006)
Klra7	killer cell lectin-like receptor, subfamily A-7	48 (p = 2.9E-06)	1.95 (p = 0.002)
**Plxdc1**	plexin domain containing 1	47 (p = 3.9E-07)	-
Kcnc2	potassium voltage gated channel, Shaw-related 2	43 (p = 4.2E-05)	-
**Duoxa1**	dual oxidase maturation factor 1	42 (p = 8.8E-06)	-
**Hsd3b2**	hydroxysteroid dehydrogenase-2, d3b2	41 (p = 5.4E-05)	4.20 (p = 0.001)
Klra22	killer cell lectin-like receptor subfamily A-22	38 (p = 1.6E-05)	1.84 (p = 0.008)
Klra15	killer cell lectin-like receptor, subfamily A-15	36 (p = 1.1E-05)	2.13 (p = 0.001)
**Gdf10**	growth differentiation factor 10	35 (p = 0.0004)	-
**Add2**	adducin 2	35 (p = 1.9E-05)	2.74 (p = 0.0002)
Klra12	killer cell lectin-like receptor subfamily A-12	35 (p = 2.1E-06)	2.06 (p = 0.008)
**Clec4e**	C-type lectin domain family 4-e	34 (p = 1.9E-05)	-

a All effects shown had p<0.05. -: no significant effect was detected.

b The gene expression level of untreated immortalizaed MEF (p32) was divided by the respective expression level of untreated primary MEF (p4).

c Cells were treated with 20 µM ascorbate 2-phosphate for 2 days before RNA harvesting. The gene expression level of vitamin C-treated immortalized MEF (p32) was divided by the respective expression level of untreated immortalized MEF (p32).

d Genes with bold letters are known to have increased expression in human cancers.

## Discussion

Mouse embryonic fibroblasts were used in this study as a model to determine the effect of vitamin C under two phenotypes: primary cells; and spontaneously immortalized cells with unlimited renewal potential. In summary, our gene expression profiling provided new evidences on the presence of unique cancer-like fingerprints in immortalized MEF and thus supports the usefulness of MEF model in evaluating phenotype-dependent and -independent effects. Additionally, our focus on the physiological level of vitamin C led to new insights since previous vitamin C studies on cells largely aimed at the pharmacological effect.

The presence of immortalization-associated genetic hallmark in immortalized embryonic fibroblasts and human cancers was previously considered [21], [22], [57]. The results of our gene expression profiling further support the relationship. Of the top 20 most over-expressed genes in immortalized MEF (Table 4), most genes belong to families that have been shown to over- express in human cancer [41]–[56]. In fact, Naalad2 belongs to the same gene family as the prostate cancer biomarker, prostate-specific antigen [43]. An increase in the inflammatory response was found during cancer development and progression [58]–[64]. Our gene expression profiling revealed an up-regulation in the inflammatory response in immortalized MEF in the absence of immune cells (Table 2, Table S2). Also observed in the absence of immune cells was the up-regulation of chemokine (C-C motif) ligand, C-type lectin domain family member, and the killer cell lectin-like receptors (Table 4), players in the innate immune response [65]–[67]. A change in the stromal-epithelial crosstalk is another hallmark during human carcinogenesis [68], [69]. As expected, in immortalized MEF, an overall enrichment of membrane-associated glycoproteins (Table 2, Table S2, S3) was found among genes that were affected (increased or decreased). Specifically, immortalization led to a down-regulation of genes belonging to the extracellular matrix functional category. Also, similar to the cancer cells [40], immortalized MEF are more sensitive to the pro-oxidant toxicity of vitamin C than the primary MEF (Fig. 1G vs. 6C). Overall, our findings support the use of primary and immortalized mouse embryonic fibroblast model pair to understand the role of physiological level vitamin C in normal and malignant cells. The utility of this model pair for other studies remains to be explored.

Scurvy is typically manifested at plasma concentration of vitamin C less than 12 µM. In this study, 5–20 µM was used to determine the physiological effects of vitamin C. Dose- and treatment duration-dependent increases in total protein and thymidine incorporation were observed in primary MEF (Fig. 1). Vitamin C is critical for normal growth [70] and similar growth promotion by vitamin C in vitro was previously reported for other primary cells [1]–[4]. This property is likely independent of the antioxidant activity of vitamin C (Fig. 1). Instead, an increase in collagen accumulation (Fig. 2) as well as an up-regulation of cell cycle and cell division-related genes (Table 1) could be the mechanisms stimulating growth. Intracellular vitamin C is needed since SVCT2−/− MEF did not respond to vitamin C treatment (Fig. 3B–E). The immortalized MEF also showed increased growth in response to physiological levels of vitamin C treatment (Fig. 6). Similar to the primary cells, a pattern of up-regulation of cell cycle- and cell division-related genes were observed (Table 3). With the massive change during immortalization (Table 2), it is not surprising that despite a similar pattern of responses in primary and immortalized MEF to the vitamin C treatment, the genes affected were not identical as shown in the Figure S3. Immortalized MEF had more genes affected by vitamin C than primary MEF (Table 1 compared to Table 3, Fig. S3). Physiological levels of vitamin C also further increased the expression of some immortalization-related genes (Tables 3 and 4), although not all. While increasing collagen in primary cells (Fig. 2), vitamin C decreased the expression of genes in the extracellular matrix functional category including some collagen genes in immortalized MEF (Table 3 and S5). This change is noteworthy as the immortalization process was found to link to a decreased extracellular matrix gene expression including some collagen genes (Table 2 and S3). The cell growth and gene expression results collectively suggest that vitamin C may not be ideal for chemoprevention. Although one allele of SVCT2 was found to be associated with a lower risk for colorectal cancer [69], the in vivo growth-promoting effect of oral vitamin C on implanted cancer cells was reported in the Gulo−/−mice that require dietary vitamin C similar to human [16]. Furthermore, the biphasic effect of vitamin C, growth promotion at the lower level and growth inhibition at higher concentration, can explain why a recent meta-analysis failed to conclude on the clinical efficacy of vitamin C in cancer treatment [14].

### Conclusions

The immortalization of mouse embryonic fibroblasts (MEF) was found to lead to similar gene expression pattern changes as in human cancer. The presence of the cancer fingerprints suggests the usefulness of the primary and immortalized MEF as a model pair in the understanding of cellular phenotype-dependent and -independent responses. As shown in cell growth analysis and gene expression profiling, intracellular vitamin C promotes the renewal of both primary and immortalized MEF. This could be the mechanism behind the in vivo anti-aging activity of vitamin C in a mouse model of Werner Syndrome, a condition characterized by premature aging [9]. Broader effects of vitamin C on growth and renewal are likely. A recent publication showed a promotion of myogenic differentiation by vitamin C under otherwise non-permissive temperature [71].

## Supporting Information

Figure S1 **Quality control of our microarray data by intensity-dependent normalization (Lowess method) and global normalization prior to the statistic analysis.**
(TIF)

Figure S2 **The correlation between qPCR and microarray in detecting the changes in the expression of six genes (r = 0.997).**
(TIF)

Figure S3 **Venn diagrams for the effect of two-day 20 µM ascorbate 2-phosphate treatment on the gene expression of primary and immortalized mouse embryonic fibroblasts.** (Top) Number of genes showed significantly (p<0.01) increased expression after vitamin C treatment. (Bottom) Number of genes showed significantly (p<0.01) decreased expression after vitamin C treatment.
(TIF)

Table S1 **Functional annotation of genes that are significantly up-regulated by vitamin C for at least 1.5 folds in primary mouse embryonic fibroblasts.**
(DOC)

Table S2 **Functional annotation of genes that are significantly up-regulated for at least 3 folds in immortalized mouse embryonic fibroblasts (MEF) compared to the primary MEF.**
(DOC)

Table S3 **Functional annotation of genes that are significantly down-regulated for at least 3 folds in immortalized mouse embryonic fibroblasts (MEF) compared to the primary MEF.**
(DOC)

Table S4 **Functional annotation of genes that are significantly up-regulated by vitamin C for at least 1.5 folds in immortalized mouse embryonic fibroblasts.**
(DOC)

Table S5 **Functional annotation of genes that are significantly down-regulated by vitamin C in immortalized mouse embryonic fibroblasts.**
(DOC)
