# Supplementary figures and images for: Cellular Phenotype-Dependent and -Independent Effects of Vitamin C on the Renewal and Gene Expression of Mouse Embryonic Fibroblasts
Source: PLoS One. 2012 Mar 13;7(3):e32957. doi: 10.1371/journal.pone.0032957 (PMC3302785; doi:10.1371/journal.pone.0032957)

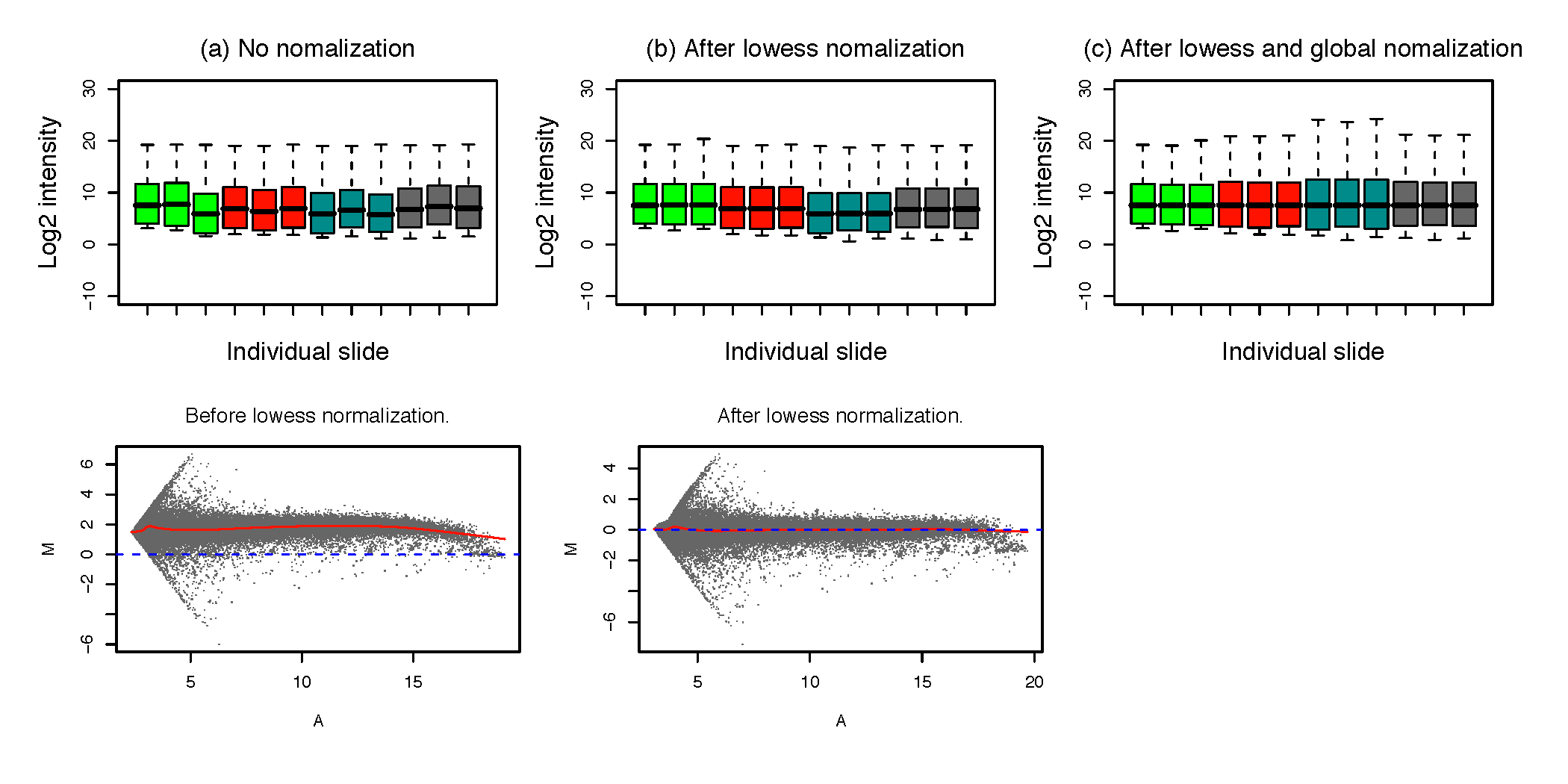

Supplement: Figure S1 — Quality control of our microarray data by intensity-dependent normalization (Lowess method) and global normalization prior to the statistic analysis. (TIF) [file pone.0032957.s001.tif]

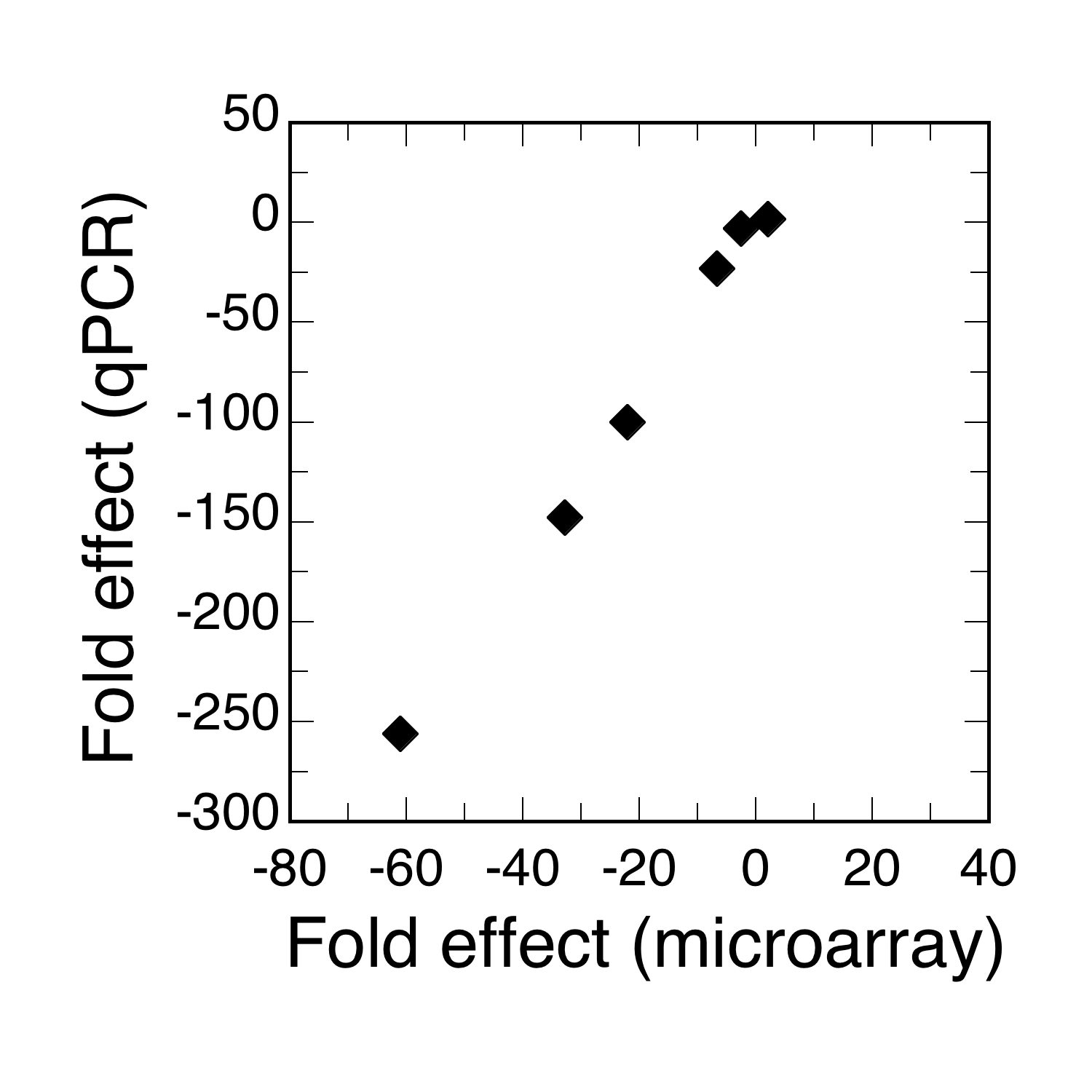

Supplement: Figure S2 — The correlation between qPCR and microarray in detecting the changes in the expression of six genes (r = 0.997). (TIF) [file pone.0032957.s002.tif]

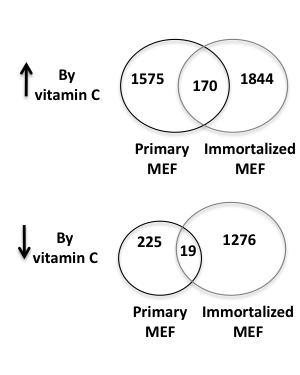

Supplement: Figure S3 — Venn diagrams for the effect of two-day 20 µM ascorbate 2-phosphate treatment on the gene expression of primary and immortalized mouse embryonic fibroblasts. (Top) Number of genes showed significantly (p<0.01) increased expression after vitamin C treatment. (Bottom) Number of genes showed significantly (p<0.01) decreased expression after vitamin C treatment. (TIF) [file pone.0032957.s003.tif]
