# Supplementary material for: Cellular Phenotype-Dependent and -Independent Effects of Vitamin C on the Renewal and Gene Expression of Mouse Embryonic Fibroblasts
Source: PLoS One. 2012 Mar 13;7(3):e32957. doi: 10.1371/journal.pone.0032957 (PMC3302785; doi:10.1371/journal.pone.0032957)
Supplement: Table S1 — Functional annotation of genes that are significantly up-regulated by vitamin C for at least 1.5 folds in primary mouse embryonic fibroblasts. (DOC) [file pone.0032957.s004.doc]

Table S1. Functional annotation of genes that are significantly up-regulated by vitamin C for at least 1.5 folds in primary mouse embryonic fibroblasts

**Enrichment of genes in cell cycle functional category fold p**

anaphase promoting complex subunit 2 1.52 0.0007

anillin, actin binding protein 1.62 0.0005

asp (abnormal spindle)-like, microcephaly associated 1.62 0.0022

cell division cycle 25 homolog C 1.55 0.0083

cyclin B1 1.66 0.0003

Rac GTPase-activating protein 1 1.56 0.0006

RAD51 homolog c 1.57 0.0075

SPC25, NDC80 kinetochore complex component, homolog 1.51 0.0059

RIKEN cDNA 4632434I11 gene 1.65 0.0078

RIKEN cDNA 6720463M24 gene 1.53 0.0091

**Enrichment of genes in cell division functional category fold p**

anaphase promoting complex subunit 2 1.52 0.0007

anillin, actin binding protein 1.62 0.0005

asp (abnormal spindle)-like, microcephaly associated 1.62 0.0022

cell division cycle 25 homolog C 1.55 0.0083

cyclin B11.66 1.66 0.0003

Rac GTPase-activating protein 1 1.56 0.0006

SPC25, NDC80 kinetochore complex component, homolog 1.51 0.0059

RIKEN cDNA 6720463M24 gene 1.53 0.0091
