# Supplementary material for: Cellular Phenotype-Dependent and -Independent Effects of Vitamin C on the Renewal and Gene Expression of Mouse Embryonic Fibroblasts
Source: PLoS One. 2012 Mar 13;7(3):e32957. doi: 10.1371/journal.pone.0032957 (PMC3302785; doi:10.1371/journal.pone.0032957)
Supplement: Table S2 — Functional annotation of genes that are significantly up-regulated for at least 3 folds in immortalized mouse embryonic fibroblasts (MEF) compared to the primary MEF. (DOC) [file pone.0032957.s005.doc]

Table S2. Functional annotation of genes that are significantly up-regulated for at least 3 folds in immortalized mouse embryonic fibroblasts (MEF) compared to the primary MEF

**Enrichment of genes in Glycoprotein functional category fold p**

a disintegrin and metallopeptidase domain 32 5.0 0.0004

a disintegrin and metallopeptidase domain 8 3.1 0.0002

a disintegrin-like and metallopeptidase with thrombospondin type 1 motif, 8 7.0 1.1E-05

a disintegrin-like and metallopeptidase with thrombospondin type 1 motif, 15 16.4 9.4E-05

a disintegrin-like and metallopeptidase with thrombospondin-like, 4 3.4 0.002

acid phosphatase-like 2 3.1 0.0003

activin A receptor, type II-like 1 8.0 0.0002

adenylate cyclase activating polypeptide 1 receptor 1 3.7 2.0E-06

adhesion molecule with Ig like domain 2 11.5 7.1E-05

alanyl (membrane) aminopeptidase 3.5 0.001

alpha fetoprotein 5.0 0.0007

angiopoietin 4 Mus musculus 5.5 0.007

angiotensin I converting enzyme (peptidyl-dipeptidase A) 2 6.6 0.002

angiotensin II receptor, type 2 11.9 2.0E-05

ATP-binding cassette, sub-family A (ABC1), member 8b 6.5 3.4E-05

ATP-binding cassette, sub-family A (ABC1), member 9 3.3 0.003

beta-site APP-cleaving enzyme 2 5.5 0.0004

betacellulin, epidermal growth factor family member 5.1 0.0006

c-fos induced growth factor 4.0 1.4E-05

c-mer proto-oncogene tyrosine 3.5 0.004

C-type lectin domain family 4, member d 6.7 2.4E-05

C-type lectin domain family 4, member e 33.7 3.7E-06

C-type lectin domain family 5, member a 6.4 0.001

cadherin 5 5.5 0.002

calcium channel, voltage-dependent, alpha2/delta subunit 3 4.8 0.0001

calreticulin 3 4.2 0.0002

cannabinoid receptor 2 4.8 0.003

carbohydrate sulfotransferase 11 4.0 0.0008

cathepsin F 3.3 3.4E-05

CD14 antigen 3.8 0.002

CD274 antigen 3.5 0.0001

CD28 antigen 3.6 0.002

CD34 antigen 17.0 2.4E-07

CD48 antigen 5.5 0.003

CD14 antigen 3.8 0.002

CD52 antigen 5.1 5.0E-05

CD53 antigen 4.7 8.5E-05

CD55 antigen 3.9 0.0003

CD80 antigen 6.2 0.001

CD84 antigen 3.3 0.0003

chemokine (C-C motif) ligand 4 7.4 8.4E-05

chemokine (C-C motif) receptor 5 3.9 9.4E-05

chemokine (C-X-C motif) ligand 16 4.0 6.5E-05

chordin-like 1 6.2 0.003

chromogranin B 3.2 0.0006

collagen, type XX, alpha 1 4.2 0.0006

collectin sub-family member 12 4.2 0.0001

colony stimulating factor 2 receptor, beta 2 4.8 0.006

complement component 1, r subcomponent 10.1 2.0E-06

complement component 1, s subcomponent 25.0 8,1E-06

complement component 2 (within H-2S) 6.9 2.8E-06

complement component 3a receptor 1 9.9 0.0004

complement factor B 4.8 0.001

contactin 1 5.2 0.003

cystatin F (leukocystatin) 14.1 0.0003

decay accelerating factor 2 4.4 0.008

decorin 6.1 0.0001

delta-like 1 homolog (Drosophila) 10.9 2.2E-05

dual oxidase maturation factor 1 42.2 8.8E-06

ecotropic viral integration site 2b 5.4 0.003

ectonucleoside triphosphate diphosphohydrolase 2 5.6 0.0002

ectonucleotide pyrophosphatase/phosphodiesterase 3 5.3 5.3E-05

ectonucleotide pyrophosphatase/phosphodiesterase 5 4.2 0.0008

endothelial-specific receptor tyrosine kinase 3.7 0.004

Eph receptor B6 4.9 6.3E-05

Epiregulin 3.4 4.3E-05

Epstein-Barr virus induced gene 3 3.1 0.0001

Fc receptor, IgG, high affinity I 3.5 0.001

Fc receptor, IgG, low affinity III 3.1 0.0005

Fc receptor, IgG, low affinity IIb 4.0 0.0004

fibroblast growth factor 10 4.5 0.0002

G protein-coupled receptor 137B 3.3 1.1E-05

G protein-coupled receptor 162 3.0 0.0002

galactosidase, alpha 3.3 0.0001

gamma-aminobutyric acid (GABA) A receptor, subunit alpha 3 3.0 0.0004

gamma-glutamyltransferase 5 8.2 0.0007

gastrin releasing peptide receptor 5.7 0.0002

glucosamine (N-acetyl)-6-sulfatase 3.1 0.0006

glutamate receptor, metabotropic 7 4.7 0.0001

glycoprotein (transmembrane) nmb 6.3 0.003

glycosyltransferase 8 domain containing 2 3.3 0.002

growth differentiation factor 10 35.4 0.0004

heparan sulfate (glucosamine) 3-O-sulfotransferase 3A1 6.8 3.6E-05

heparan sulfate (glucosamine) 3-O-sulfotransferase 3B1 4.3 8.6E-05

hepatitis A virus cellular receptor 2 4.7 0.0006

histocompatibility 2, class II, locus Mb1 7.1 9.3E-06

hydroxysteroid 11-beta dehydrogenase 1 3.2 0.0003

immunoglobulin superfamily containing leucine-rich repeat 6.4 0.0003

insulin-like growth factor binding protein 6 4.0 6.6E-06

integrin alpha X 15.5 8.0E-05

integrin beta 2 3.3 8.7E-06

intercellular adhesion molecule 1 3.1 0.0008

interleukin 1 receptor-like 1 4.0 0.0002

interleukin 15 17.8 1.5E-05

interleukin 18 receptor 1 7.2 0.0002

interleukin 2 receptor, beta chain 3.2 0.003

interleukin 7 receptor 5.4 0.0004

junction adhesion molecule 2 6.2 8.2E-05

keratocan 23.3 9.6E-06

killer cell lectin-like receptor, subfamily A, member 12 34.7 1.9E-05

killer cell lectin-like receptor, subfamily A, member 2 9.7 7.5E-05

killer cell lectin-like receptor, subfamily A, member 3 28.7 0.003

killer cell lectin-like receptor, subfamily A, member 22 38.3 1.6E-05

killer cell lectin-like receptor, subfamily A, member 7 48.0 2.9E-06

killer cell lectin-like receptor, subfamily D, member 1 3.6 0.001

kit ligand 5.7 0.007

leukocyte-associated Ig-like receptor 1 4.0 0.002

leukocyte immunoglobulin-like receptor, subfamily B, member 4 7.1 4.2E-05

lipoprotein lipase 6.3 0.0002

LMBR1 domain containing 1 4.0 0.0002

lumican 3.5 0.0001

lymphocyte antigen 6 complex, locus A 6.0 4.1E-06

lymphocyte antigen 6 complex, locus C2 9.5 3.4E-05

lymphocyte antigen 6 complex, locus F 11.8 6.9E-05

lymphocyte antigen 6 complex, locus I 4.5 2.3E-06

Ly6/neurotoxin 1 4.2 0.0002

mannosidase 1, alpha 4.0 0.0009

matrix metallopeptidase 12 9.7 0.0001

matrix metallopeptidase 2 3.0 0.0002

matrix metallopeptidase 3 3.8 3.0E-06

matrix metallopeptidase 8 8.5 0.0006

microfibrillar associated protein 5 6.4 0.0001

multiple EGF-like-domains 10 4.4 0.0001

N-acetylated alpha-linked acidic dipeptidase 2 57.6 2.9E-05

netrin 1 3.9 0.006

neuronal growth regulator 1 3.0 0.0004

neuropeptide Y receptor Y1 5.1 0.0008

Nfat activating molecule with ITAM motif 1 3.7 0.001

oncoprotein induced transcript 3 5.2 0.0003

paired immunoglobin-like type 2 receptor alpha 9.6 0.0008

paired immunoglobin-like type 2 receptor beta 1 3.8 0.002

patched homolog 2 5.9 7.7E-05phosphatidic acid phosphatase type 2B 5.7 1.4E-05

phosphatidic acid phosphatase type 2C 3.1 0.001

platelet-derived growth factor, D polypeptide 3.3 0.003

plexin domain containing 1 47.3 3.9E-07

potassium inwardly-rectifying channel, subfamily J, member 3 3.5 0.001

potassium voltage gated channel, Shaw-related subfamily, member 2 42.7 4.2E-05

prokineticin receptor 1 8.6 0.002

proline arginine-rich end leucine-rich repeat 6.1 0.0008

prominin 1 3.6 0.002

proprotein convertase subtilisin/kexin type 1 3.8 5.7E-06

prostaglandin E receptor 4 (subtype EP4) 4.9 0.001

prostaglandin I receptor (IP) 4.2 0.0001

prostaglandin-endoperoxide synthase 1 4.4 4.6E-05

protein tyrosine phosphatase, receptor type, C 3.0 0.0004

protein tyrosine phosphatase, receptor type, J 3.6 8.2E-06

protocadherin 21 3.3 0.008

protocadherin beta 14 8.0 3.9E-05

protogenin homolog 5.4 0.0008

regulator of G-protein signaling 20 9.0 0.0001

reticulon 4 receptor-like 1 3.8 0.0003

retinoic acid early transcript 1E 3.5 1.8E-05

secreted frizzled-related protein 1 6.1 0.0001

selectin, endothelial cell 8.0 0.0001

selenoprotein P, plasma, 1 6.8 1.5E-05

serine (or cysteine) peptidase inhibitor, clade B, member 2 4.0 0.004

serine (or cysteine) peptidase inhibitor, clade G, member 1 3.1 0.001

signal-regulatory protein alpha 3.1 0.002

sodium channel, voltage-gated, type III, beta 3.1 0.003

solute carrier family 15, member 3 5.7 0.0002

solute carrier family 2 (facilitated glucose transporter), member 5 3.2 0.0006

solute carrier family 22 (organic cation transporter), member 2 31.1 9.6E-05

solute carrier family 22 (organic cation transporter), member 4 8.0 1.5E-06

solute carrier family 4 (anion exchanger), member 4 5.0 2.2E-05

solute carrier family 44, member 3 7.3 0.0007

ST3 beta-galactoside alpha-2,3-sialyltransferase 1 3.1 8.7E-05

ST8 alpha-N-acetyl-neuraminide alpha-2,8-sialyltransferase 4 6.8 0.003

stabilin 1 4.1 1.1E-05

sushi, nidogen and EGF-like domains 1 3.2 0.0009

synaptoporin 3.6 0.002

synaptotagmin I 11.1 0.0007

tachykinin receptor 2; similar to substance K receptor 6.6 0.008

thrombopoietin 5.5 0.001

toll-like receptor 1 6.7 0.0004

toll-like receptor 2 4.1 0.0001

toll-like receptor 3 5.9 3.6E-05

toll-like receptor 7 4.4 0.0003

transforming growth factor, beta receptor III 4.2 0.0002

transmembrane protein 8 (five membrane-spanning domains) 4.9 0.0003

TRH-degrading enzyme 6.2 0.005

triggering receptor expressed on myeloid cells 2 3.0 0.0004

tumor necrosis factor (ligand) superfamily, member 12 3.0 4.2E-05

tumor necrosis factor (ligand) superfamily, member 13b 3.4 0.0005

tumor necrosis factor receptor superfamily, member 1b 3.2 0.0008

tumor necrosis factor receptor superfamily, member 26 9.7 2.4E-05

UDP-N-acetyl-alpha-D-galactosamine:polypeptide 5.6 0.001

N-acetylgalactosaminyltransferase 13

unc-93 homolog B1 4.0 0.0002

xanthine dehydrogenase 12.4 0.0006

**Enrichment of genes in Inflammatory response functional category fold p**

arachidonate 5-lipoxygenase 3.4 0.0001

cannabinoid receptor 2 (macrophage) 4.8 0.003

CD14 antigen 3.8 0.002

CD55 antigen 3.9 0.0003

chemokine (C-C motif) ligand 12 4.4 3.1E-05

chemokine (C-C motif) ligand 3 17.1 1.8E-05

chemokine (C-C motif) ligand 4 7.4 8.4E-05

chemokine (C-C motif) receptor 5 3.9 9.4E-05

chemokine (C-X-C motif) ligand 10 5.9 0.003

complement component 1, r subcomponent 10.1 1.9E-06

complement component 1, s subcomponent 25.0 8.1E-06

complement component 2 (within H-2S) 6.9 2.8E-06

complement factor B 4.8 0.001

decay accelerating factor 2 4.4 0.008

Fc receptor, IgG, high affinity I 3.5 0.001

Fc receptor, IgG, low affinity III 3.1 0.0005

gamma-glutamyltransferase 5 8.1 0.0007

integrin beta 2 3.3 8.7E-06

intercellular adhesion molecule 1 3.1 0.0008

neutrophil cytosolic factor 1 4.0 0.001

peroxisome proliferator activated receptor gamma 5.7 4.0E-05

serine (or cysteine) peptidase inhibitor, clade B, member 1a 5.9 0.0001

serine (or cysteine) peptidase inhibitor, clade B, member 1b 5.0 6.6E-05

serine (or cysteine) peptidase inhibitor, clade B, member 1c 6.5 0.0007

serine (or cysteine) peptidase inhibitor, clade B, member 2 4.0 0.004

serine (or cysteine) peptidase inhibitor, clade G, member 1 3.1 0.001

serum amyloid A 3 6.8 1.7E-06

stabilin 1 4.1 1.1E-05

toll-like receptor 1 6.7 0.0004

toll-like receptor 2 4.1 0.0001

toll-like receptor 3 5.9 3.6E-05

toll-like receptor 7 4.4 0.0003

tumor necrosis factor receptor superfamily, member 1b 3.1 0.0008
