# Supplementary material for: Cellular Phenotype-Dependent and -Independent Effects of Vitamin C on the Renewal and Gene Expression of Mouse Embryonic Fibroblasts
Source: PLoS One. 2012 Mar 13;7(3):e32957. doi: 10.1371/journal.pone.0032957 (PMC3302785; doi:10.1371/journal.pone.0032957)
Supplement: Table S3 — Functional annotation of genes that are significantly down-regulated for at least 3 folds in immortalized mouse embryonic fibroblasts (MEF) compared to the primary MEF. (DOC) [file pone.0032957.s006.doc]

Table S3. Functional annotation of genes that are significantly down-regulated for at least 3 folds in immortalized mouse embryonic fibroblasts (MEF) compared to the primary MEF

**Enrichment of genes in Glycoprotein functional category fold p**

5-hydroxytryptamine (serotonin) receptor 1B

a disintegrin and metallopeptidase domain 12 3.1 2.9E-05

a disintegrin-like and metallopeptidase with thrombospondin-like 2 15.2 4.7E-05

a disintegrin-like and metallopeptidase with thrombospondin type 1 motif, 16 13.3 4.8E-05

acid phosphatase 5, tartrate resistant 7.1 1.6E-07

adenosine A1 receptor 3.4 0.009

adenylate cyclase 2 4.4 0.004

adenylate cyclase 5; similar to adenylate cyclase 5 3.4 0.005

adrenergic receptor, alpha 1b 5.8 4.2E-05

aggrecan 12.0 1.4E-05

agrin 3.0 0.0003

angiopoietin-like 6 3.8 0.002

angiotensin II receptor, type 1a 5.9 0.001

aquaporin 5 22.2 1.34E-5

artemin 4.5 0.0001

asporin 4.1 0.0001

blood vessel epicardial substance 48.2 1.5E-05

bone morphogenetic protein 4 11.7 4.8E-06

bone morphogenetic protein 5 3.6 0.003

bone morphogenetic protein 8b 3.9 0.003

brevican 3.7 0.0006

C-type lectin domain family 4, member a2 3.5 0.002

cadherin 10 27.4 5.7E-06

cadherin 13 11.8 4.6E-06

cadherin 15 10.6 0.0005

cadherin 3 3.6 0.002

cadherin 6 3.8 0.002

cadherin 8 10.0 0.0002

cadherin, EGF LAG seven-pass G-type receptor 1 6.1 0.0006

calcium channel, voltage-dependent, T type, alpha 1H subunit 22.5 5.3E-05

calsyntenin 2 3.2 0.0008

carbohydrate (N-acetylglucosamine 6-O) sulfotransferase 5 3.9 0.001

carbohydrate (N-acetylglucosamino) sulfotransferase 7 15.8 2.1E-07

carbohydrate sulfotransferase 2 5.1 0.005

carbonic anhydrase 6 13.3 5.6E-05

carbonic anyhydrase 12 4.0 0.003

carboxylesterase 1 6.9 0.0003

carboxypeptidase A4 3.2 0.001

carboxypeptidase N, polypeptide 2 4.2 0.0005

carboxypeptidase Z 3.5 0.003

catenin (cadherin associated protein), delta 2 18.0 5.5E-06

cathepsin C 10.4 1.7E-06

CD200 antigen 5.0 0.0009

CD97 antigen 3.8 2.7E-05

cell adhesion molecule with homology to L1CAM 6.8 9.3E-05

cerebellin 1 precursor protein; similar to precerebellin-1 12.4 0.0004

chemokine (C-X-C motif) receptor 6 4.6 0.0009

chondrolectin 20.4 0.0006

ciliary neurotrophic factor receptor 4.2 0.0004

coagulation factor C homolog 45.1 1.1E-05

coagulation factor II (thrombin) receptor-like 1 5.9 0.0002

collagen, type VII, alpha 1 3.3 6.5E-06

collagen, type XI, alpha 1 5.5 0.0002

collagen, type XII, alpha 1 6.0 3.1E-05

collagen, type XIV, alpha 1 4.4 0.002

collagen, type XXIV, alpha 1 21.6 9.9E-05

collagen, type XXVII, alpha 1 3.6 0.0009

coxsackie virus and adenovirus receptor 7.6 1.2E-05

cysteine-rich secretory protein LCCL domain containing 2 42.6 6.0E-08

cytochrome b reductase 1 3.2 0.004

cytokine receptor-like factor 1 5.6 1.4E-05

deleted in colorectal carcinoma 4.1 0.0006

delta/notch-like EGF-related receptor 5.1 0.0002

dickkopf homolog 1 49.4 1.8E-05

dipeptidase 1 6.4 1.7E-05

DNA segment, Chr 3, Brigham & Women's Genetics 0562 expressed 3.0 0.001

endoglin 7.3 9.2E-07

endomucin 3.1 5.2E-05

EMI domain containing 2 8.5 0.0002

Eph receptor A3 24.4 0.0009

Eph receptor A5 17.9 9.0R-05

Eph receptor B2 4.2 0.0009

epidermal growth factor-containing fibulin-like ECM protein 1 8.5 2.5E-05

estrogen receptor 2 (beta) 3.3 0.001

fetuin beta 3.1 2.8E-05

fibroblast growth factor 18 3.5 0.0005

fibroblast growth factor 5 30.1 1.9E-05

fibroblast growth factor 9 5.5 8.3E-05

fibroblast growth factor receptor 2 4.9 0.0006

fibronectin type III domain containing 5 3.1 0.001

FMS-like tyrosine kinase 1 3.2 0.0001

Fras1 related extracellular matrix protein 2 3.1 0.005

Fraser syndrome 1 homolog 5.0 1.0E-05

frizzled homolog 10 5.8 0.0003

G protein-coupled receptor 1 3.8 0.0004

G protein-coupled receptor 17 4.1 0.0007

gamma-aminobutyric acid (GABA) A receptor, subunit alpha 1 5.7 0.0004

gamma-aminobutyric acid (GABA) A receptor, subunit beta 3 7.4 0.0004

glutamate receptor, ionotropic, delta 2 7.6 0.0005

glutamate receptor, ionotropic, kainate 1 5.3 4.1E-05

glutamyl aminopeptidase 18.3 7.6E-05

glypican 1 4.5 6.8E-05

glypican 4 3.5 1.2E-05

granzyme B 4.4 0.002

granzyme E 5.0 0.0004

growth differentiation factor 15 3.7 3.0E-05

heparan sulfate 6-O-sulfotransferase 1 3.1 0.0002

hephaestin 3.3 0.0001

histamine receptor H1 3.4 2.4E-05

insulin-like growth factor binding protein 2 60.2 1.7E-05

inter-alpha trypsin inhibitor, heavy chain 2 4.0 0.003

interleukin 1 receptor antagonist 4.9 5.3E-05

interleukin 13 receptor, alpha 1 3.5 4.0E-05

interleukin 20 receptor, alpha 22.2 0.0002

jagged 1 7.9 0.0002

kallikrein related-peptidase 8 7.6 1.1E-05

kelch domain containing 7A 3.4 7.6E-07

keratin 8 4.0 3.2E-05

keratin 18 79.2 7.3E-07

laminin, alpha 1 8.1 0.002

leprecan 1 3.3 0.0001

leucine rich repeat containing 15 3.7 0.0004

leucine rich repeat containing G protein coupled receptor 5 6.2 8.7E-06

leucine rich repeat protein 1, neuronal 14.6 2.0E-07

leucine-rich repeat LGI family, member 2 4.8 6.3E-05

leucine-rich repeat LGI family, member 3 3.7 0.0007

LY6/PLAUR domain containing 6 3.6 0.001

major histocompatibility complex, class I-related 3.4 6.2E-06

MANSC domain containing 1 12.2 4.0E-07

mast cell protease 8 5.4 0.003

matrix metallopeptidase 13 6.6 7.4E-05

matrix metallopeptidase 23 26.0 7.5E-08

matrix metallopeptidase 9 12.6 9.2E-05

melanoma cell adhesion molecule 19.2 3.9E-06

microfibrillar-associated protein 4 16.5 4.3E-05

multiple EGF-like-domains 6 125.1 5.1E-06

NEL-like 2 6.5 5.6E-05

nephroblastoma overexpressed gene 3.2 0.002

nerve growth factor receptor (TNFR superfamily, member 16) 4.0 8.6E-05

netrin 4 4.8 0.0009

neuritin 1 5.6 8.1E-05

neuronal pentraxin 2 20.6 0.0001

neuropeptide Y receptor Y2 23.8 2.1E-05

neurotrophic tyrosine kinase, receptor, type 2 8.5 0.0001

neurotrophin 3 4.8 4.2E-05

NIPA-like domain containing 2 8.8 0.0001

noggin 6.1 0.004

odd Oz/ten-m homolog 2 4.7 0.001

odd Oz/ten-m homolog 3 15.2 5.6E-05

olfactomedin 1 4.5 3.8E-05

olfactomedin 3 6.8 2.6E-06

opsin 3 3.0 8.2E-05

opsin 4 (melanopsin) 8.1 0.0003

parathyroid hormone 1 receptor 9.6 5.1E-05

patatin-like phospholipase domain containing 3 14.2 2.3E-05

pentraxin related gene 4.2 2.5E-06

phospholipase A2, group VII (platelet-activating factor acetylhydrolase) 7.9 2.4E-06

plexin B1 3.6 0.002

plexin C1 3.8 0.007

potassium channel, subfamily K, member 1 21.6 0.002

potassium large conductance calcium-activated channel, subfamily M, beta 17.5 4.3E-06

member 1

potassium large conductance calcium-activated channel, subfamily M, beta 8.7 0.0002

member 4

potassium voltage-gated channel, Isk-related family, member 1-like 6.2 4.1E-09

potassium voltage-gated channel, shaker-related subfamily, member 1 7.3 0.0002

pregnancy-associated plasma protein A 8.2 4.6E-05

pro-opiomelanocortin-alpha 8.2 4.1E-06

prokineticin receptor 2 3.2 0.006

prolactin receptor 6.1 0.0002

proprotein convertase subtilisin/kexin type 5 20.1 1.6E-05

proprotein convertase subtilisin/kexin type 9 40.3 4.6E-06

prostaglandin E receptor 3 (subtype EP3) 8.0 0.0003

protease, serine, 12 neurotrypsin 11.9 4.8E-05

protease, serine, 16 3.4 0.0005

protease, serine, 35 3.0 0.001

protein tyrosine phosphatase, receptor type, B 10.1 1.1E-05

protein tyrosine phosphatase, receptor type, D 8.0 0.0003

protein tyrosine phosphatase, receptor type, F 7.7 1.3E-05

protein tyrosine phosphatase, receptor type, U 4.9 0.0002

protocadherin 20 18.2 1.2E-06

R-spondin 3 homolog (Xenopus laevis) 6.6 0.0004

R-spondin homolog (Xenopus laevis) 6.1 0.0006

reelin 3.3 0.004

regenerating islet-derived 1 7.7 0.0001

rhodopsin 3.2 0.001

ring finger protein 128 8.2 5.4E-05

roundabout homolog 1 5.8 2.5E-06

sclerostin domain containing 1 5.5 0.0003

secreted phosphoprotein 1 6.3 4.0E-06

sema domain, immunoglobulin domain (Ig), TM domain, and short 4.7 0.006

cytoplasmic domain

sema domain, immunoglobulin domain (Ig), short basic domain, secreted, 3.6 7.9E-05

(semaphorin) 3E

sema domain, immunoglobulin domain (Ig), short basic domain, secreted, 3.5 0.0004

(semaphorin) 3F

serine protease inhibitor, Kunitz type 2 7.8 0.0001

shadow of prion protein 3.1 0.004

signal peptide, CUB domain, EGF-like 1 6.5 0.0006

Lymphocyte antigen 6H precursor (Ly-6H) 3.5 0.001

fibrillin 2 9.0 2.8E-05

SPARC related modular calcium binding 1 18.3 1.8E-06

SPARC-like 1 5.4 0.001

solute carrier family 7 (cationic amino acid transporter, y+ system), member 5 3.4 9.8E-05

solute carrier family 1 (high affinity aspartate/glutamate transporter), member 3.7 8.7E-07

solute carrier family 12, member 1 3.1 0.0003

solute carrier family 15 (H+/peptide transporter), member 2 4.5 0.003

solute carrier family 2 (facilitated glucose transporter), member 13 18.8 0.001

solute carrier family 2 (facilitated glucose transporter), member 3 21.7 0.0002

solute carrier family 2 (facilitated glucose transporter), member 4 39.2 1.7E-06

solute carrier family 38, member 1 5.0 9.8E-06

solute carrier family 38, member 4 15.5 6.2E-06

solute carrier family 43, member 1 33.0 1.0E-05

solute carrier family 5 (choline transporter), member 7 13.0 0.0002

solute carrier family 6 (neurotransmitter transporter, dopamine), member 3 3.4 0.0005

solute carrier family 7 (cationic amino acid transporter, y+ system), member 3 7.3 0.0003

solute carrier organic anion transporter family, member 4a1 3.7 0.0001

sortilin-related VPS10 domain containing receptor 2 3.8 5.4E-05

sparc/osteonectin, cwcv and kazal-like domains proteoglycan 3 3.4 0.0001

sperm acrosome associated 1 27.9 2.3E-05

ST6 (alpha-N-acetyl-neuraminyl-2,3-beta-galactosyl-1,3)-N- 37.3 2.4E-05

acetylgalactosaminide alpha-2,6-sialyltransferase 3

ST6 (alpha-N-acetyl-neuraminyl-2,3-beta-galactosyl-1,3)-N- 3.5 0.006

acetylgalactosaminide alpha-2,6-sialyltransferase 5

stanniocalcin 1 5.2 5.9E-06

succinate receptor 1 4.9 0.001

sushi domain containing 2 5.5 0.003

synaptic vesicle glycoprotein 2 b 3.5 0.0004

synaptotagmin VIII 4.7 0.002

tenascin C 3 0.001

tenascin N 9.3 0.0002

tenomodulin 95.6 7.9E-05

tetraspanin 18 22.5 1.6E-05

tetraspanin 2 9.2 0.0008

thrombospondin 4 11.4 0.0003

thromboxane A2 receptor 4.1 2.4E-05

thyroglobulin 4.9 2.2E-06

tissue factor pathway inhibitor 5.1 0.0004

tissue factor pathway inhibitor 2 20.8 2.1E-05

transferrin receptor 2 11.6 0.0001

transforming growth factor, beta 2 4.2 9.0E-06

transforming growth factor, beta 3 7.3 8.4E-05

transient receptor potential cation channel, subfamily V, member 2 3.7 0.0001

transmembrane channel-like gene family 6 5.4 4.6E-05

transmembrane protein 132E 38.1 1.8E-05

transmembrane protein 30B 15.5 5.0E-05

transmembrane protein with EGF-like and two follistatin-like domains 1 4.2 0.001

transmembrane protein with EGF-like and two follistatin-like domains 2 3.2 0.0002

tubulointerstitial nephritis antigen-like 1 3.2 9.0E-05

tumor necrosis factor (ligand) superfamily, member 11 29.6 1.7E-05

tumor necrosis factor (ligand) superfamily, member 8 3.3 0.0005

tumor necrosis factor receptor superfamily, member 11b (osteoprotegerin) 3.4 0.0001

tumor necrosis factor receptor superfamily, member 19 5.2 0.004

UDP glucuronosyltransferase 2 family, polypeptide A2 3.2 0.006

UDP-GlcNAc:betaGal beta-1,3-N-acetylglucosaminyltransferase 5 25.2 4.4E-05

UDP-N-acetyl-alpha-D-galactosamine:polypeptide N- 12.6 0.0002

acetylgalactosaminyltransferase 3

unc-5 homolog B 7.9 3.2E-05

unc-5 homolog D 3.0 0.006

uroplakin 1B 29.3 3.2E-05

uroplakin 3B 23.5 2.9E-05

vanin 1 6.4 5.2E-05

vomeronasal 1 receptor, B3 3.1 0.0002

wingless related MMTV integration site 10b 3.6 0.003

wingless-related MMTV integration site 11 6.1 7.5E-06

wingless-related MMTV integration site 2 7.9 1.4E-05

wingless-related MMTV integration site 4 12.0 5.8E-07

WNT1 inducible signaling pathway protein 1 3.6 0.0002

WNT1 inducible signaling pathway protein 2 10.9 5.64E-06

**Enrichment of genes in Extracellular matrix functional category fold p**

a disintegrin and metallopeptidase domain 12 (meltrin alpha) 3.0 2.9E-05

a disintegrin-like and metallopeptidase with thrombospondin-like 2 15.2 4.7E-05

a disintegrin-like and metallopeptidase (reprolysin type) with thrombospondin 13.3 4.8E-05

type 1 motif, 16

aggrecan 12.0 1.4E-05

agrin 3.0 0.0003

asporin 4.1 0.0002

bone morphogenetic protein 4 9.9 0.0004

brevican 3.7 0.0006

carboxypeptidase Z 3.5 0.003

cell adhesion molecule with homology to L1CAM 4.1 0.0002

collagen, type VII, alpha 1 3.3 6.5E-06

collagen, type VIII, alpha 2 5.6 8.6E-06

collagen, type XI, alpha 1 5.5 0.0002

collagen, type XII, alpha 1 6.0 3.1E-05

collagen, type XIV, alpha 1 4.4 0.002

collagen, type XXIV, alpha 1 21.6 9.9E-05

collagen, type XXVII, alpha 1 3.6 0.0009

connective tissue growth factor 3.1 5.4E-05

cysteine-rich secretory protein LCCL domain containing 2 42.6 6.0E-08

desmin 33.3 0.0003

EGF-like, fibronectin type III and laminin G domains 3.1 0.001

elastin 3.5 0.002

EMI domain containing 2 8.5 0.002

Fras1 related extracellular matrix protein 2 3.1 0.005

Fraser syndrome 1 homolog 5.0 1.0E-05

glypican 1 4.5 6.8E-05

glypican 4 3.5 1.2E-05

laminin, alpha 1 8.1 0.002

leprecan 1 3.3 0.0001

matrix metallopeptidase 13 6.6 7.3E-05

matrix metallopeptidase 23 26.0 7.5E-08

matrix metallopeptidase 9 8.1 8.0E-05

microfibrillar-associated protein 4 16.4 4.3E-05

nephronectin 6.6 0.0006

netrin 4 5.6 8.2E-05

reelin 3.3 0.004

similar to fibrillin 2; fibrillin 2 9.0 2.8E-05

SPARC-like 1 5.4 0.001

SPARC related modular calcium binding 1 18.3 1.8E-06

sparc/osteonectin, cwcv and kazal-like domains proteoglycan 3 3.4 0.0001

tenascin C 3.0 0.001

tenascin N 9.2 0.0002

transforming growth factor, beta 3 7.3 8.4E-05

tumor necrosis factor receptor superfamily, member 11b (osteoprotegerin) 3.4 0.0001

wingless related MMTV integration site 10b 3.6 0.003

wingless-related MMTV integration site 11 6.1 7.5E-06

wingless-related MMTV integration site 2 7.9 1.4E-05

wingless-related MMTV integration site 4 12.0 5.8E-07
