# Supplementary material for: Cellular Phenotype-Dependent and -Independent Effects of Vitamin C on the Renewal and Gene Expression of Mouse Embryonic Fibroblasts
Source: PLoS One. 2012 Mar 13;7(3):e32957. doi: 10.1371/journal.pone.0032957 (PMC3302785; doi:10.1371/journal.pone.0032957)
Supplement: Table S4 — Functional annotation of genes that are significantly up-regulated by vitamin C for at least 1.5 folds in immortalized mouse embryonic fibroblasts. (DOC) [file pone.0032957.s007.doc]

Table S4. Functional annotation of genes that are significantly up-regulated by vitamin C for at least 1.5 folds in immortalized mouse embryonic fibroblasts

**Enrichment of genes in cell cycle functional category fold p**

acidic (leucine-rich) nuclear phosphoprotein 32 family, member B 1.5 0.005

aryl-hydrocarbon receptor 1.9 0.006

asp (abnormal spindle)-like, microcephaly associated 2.1 0.005

aurora kinase A 1.9 0.004

breast cancer 2 1.5 0.004

budding uninhibited by benzimidazoles 1 homolog 1.9 0.001

budding uninhibited by benzimidazoles 1 homolog, beta 1.8 0.0005

cell division cycle 25 homolog A 1.7 0.005

cell division cycle 25 homolog B 1.5 0.006

cell division cycle 25 homolog C 1.9 0.0008

cell division cycle 45 homolog-like 1.7 9.6E-05

cell division cycle 6 homolog 1.7 0.002

cell division cycle associated 2 1.8 0.001

cell division cycle associated 3 1.8 0.009

cell division cycle associated 5 1.8 0.006

cell division cycle associated 8 1.8 0.002

centromere protein E 1.9 0.002

centrosomal protein 55 1.9 0.006

checkpoint kinase 1 homolog 1.7 0.004

chromatin assembly factor 1, subunit A (p150) 2.0 0.003

chromatin assembly factor 1, subunit B (p60) 2.1 0.004

claspin homolog 1.7 0.007

CLIP associating protein 1 1.5 0.004

coiled-coil domain containing 99 1.8 0.004

CTF18, chromosome transmission fidelity factor 18 homolog 2.1 0.006

cyclin A1 1.6 0.009

cyclin A2 1.6 0.008

cyclin B1 1.8 0.008

cyclin D1 1.9 0.003

cyclin F 1.8 0.0008

cyclin-dependent kinase 6 1.7 0.0002

cyclin-dependent kinase inhibitor 3 1.6 0.003

DBF4 homolog 1.8 0.007

DnaJ (Hsp40) homolog, subfamily C, member 2 2.0 0.003

discs, large (Drosophila) homolog-associated protein 5 1.7 0.0002

E2F transcription factor 2 1.7 0.005

establishment of cohesion 1 homolog 2 1.6 0.0008

excision repair cross-complementing rodent repair deficiency 1.5 0.008

complementation group 6 - like

extra spindle poles-like 1 2.2 0.002

Fanconi anemia, complementation group A 1.9 0.004

Fanconi anemia, complementation group D2 1.7 0.003

helicase, lymphoid specific 1.8 0.006

high mobility group AT-hook 2 2.1 0.002

inner centromere protein 1.7 0.0008

kinesin family member 11 2.2 0.006

kinesin family member 18A 1.8 0.0004

kinetochore associated 1 1.8 0.0002

Mdm2, transformed 3T3 cell double minute p53 binding protein 2.1 0.002

meiosis-specific nuclear structural protein 1 2.2 0.008

minichromosome maintenance deficient 2 mitotin 1.7 0.0005

minichromosome maintenance deficient 3 2.0 0.001

minichromosome maintenance deficient 7 1.6 0.0008

minichromosome maintenance deficient 8 1.5 0.002

mitogen-activated protein kinase 4 2.8 0.008

NIMA (never in mitosis gene a)-related expressed kinase 2 2.0 0.003

nuclear autoantigenic sperm protein (histone-binding) 2.3 0.003

nuclear distribution gene C homolog 1.6 0.006

par-6 (partitioning defective 6) homolog beta 1.7 0.005

3-phosphoglycerate dehydrogenase 2.6 0.001

polo-like kinase 1 1.6 0.004

polymerase (DNA directed), alpha 1 1.8 0.003

proteasome 26S subunit, ATPase 3, interacting protein 1.8 0.002

protein kinase, membrane associated tyrosine/threonine 1 1.6 0.009

RAD51 homolog 1.6 0.008

regulator of chromosome condensation 1 2.0 0.001

regulator of chromosome condensation 2 1.6 0.008

replication protein A1 1.6 0.001

ribosomal protein S6 kinase, polypeptide 2 1.8 0.001

Rap1 interacting factor 1 homolog (yeast) 1.7 0.0003

septin 3 3.3 0.004

SET domain containing (lysine methyltransferase) 8 1.6 0.0001

shugoshin-like 1 1.7 0.003

sperm associated antigen 5 1.8 0.001

spindle assembly 6 homolog 1.7 0.006

stathmin 1 1.8 1.004

structural maintenance of chromosomes 2 2.2 0.007

SUMO/sentrin specific peptidase 5 1.7 0.002

suppressor of variegation 3-9 homolog 1 1.5 0.0003

suppressor of variegation 3-9 homolog 2 2.2 0.007

timeless homolog 1.7 6.8E-05

TPX2, microtubule-associated protein homolog 1.6 0.002

ubiquitin-like, containing PHD and RING finger domains, 1 1.6 0.009

vomeronasal 2, receptor 53 1.8 0.0005

ZW10 interactor 1.7 0.006

**Enrichment of genes in cell division functional category fold p**

asp (abnormal spindle)-like, microcephaly associated 2.1 0.005

breast cancer 2 1.5 0.004

budding uninhibited by benzimidazoles 1 homolog 1.9 0.001

budding uninhibited by benzimidazoles 1 homolog, beta 1.8 0.0006

cell division cycle 25 homolog A 1.7 0.005

cell division cycle 25 homolog B 1.5 0.006

cell division cycle 25 homolog C 1.9 0.0008

cell division cycle 45 homolog-like 1.7 9.6E-05

cell division cycle 6 homolog 1.7 0.002

cell division cycle associated 2 1.7 0.004

cell division cycle associated 3 1.8 0.009

cell division cycle associated 5 1.8 0.002

cell division cycle associated 7 2.0 0.005

cell division cycle associated 8 1.9 0.002

centromere protein E 1.9 0.002

centrosomal protein 55 1.9 0.006

CLIP associating protein 1 1.6 0.003

coiled-coil domain containing 99 1.8 0.0009

cyclin A1 1.7 0.009

cyclin A2 1.6 0.008

cyclin B1 1.8 0.008

cyclin D1 1.9 0.001

cyclin F 1.8 0.0008

cyclin-dependent kinase 6 1.7 0.0002

excision repair cross-complementing rodent repair deficiency, 1.8 0.0001

complementation group 6 - like

excision repair cross-complementing rodent repair deficiency, 1.5 0.002

complementation group 2

helicase, lymphoid specific 1.8 0.006

high mobility group AT-hook 2 1.9 0.0008

inner centromere protein 1.7 0.0008

kinesin family member 11 2.2 0.006

kinetochore associated 1 1.8 0.0002

minichromosome maintenance deficient 5, cell division cycle 46 1.9 0.0002

NIMA (never in mitosis gene a)-related expressed kinase 2 2.0 0.003

nuclear distribution gene C homolog 1.6 0.006

par-6 (partitioning defective 6) homolog beta 1.7 0.005

phosphatidylinositol 3-kinase, catalytic, beta polypeptide 1.7 0.003

polo-like kinase 1 1.6 0.004

regulator of chromosome condensation 1 2.0 0.001

regulator of chromosome condensation 2 1.6 0.008

SET domain containing (lysine methyltransferase) 8 1.6 0.0001

shugoshin-like 1 1.7 0.003

sperm associated antigen 5 1.8 0.001

structural maintenance of chromosomes 2 2.2 0.007

SUMO/sentrin specific peptidase 5 1.7 0.002

timeless homolog 1.7 6.8E-05

topoisomerase (DNA) II alpha 2.1 0.0008

vomeronasal 2, receptor 53 1.8 0.0005

ZW10 interactor 1.7 0.006
