# Supplementary material for: Cellular Phenotype-Dependent and -Independent Effects of Vitamin C on the Renewal and Gene Expression of Mouse Embryonic Fibroblasts
Source: PLoS One. 2012 Mar 13;7(3):e32957. doi: 10.1371/journal.pone.0032957 (PMC3302785; doi:10.1371/journal.pone.0032957)
Supplement: Table S5 — Functional annotation of genes that are significantly down-regulated by vitamin C in immortalized mouse embryonic fibroblasts. (DOC) [file pone.0032957.s008.doc]

Table S5. Functional annotation of genes that are significantly down-regulated by vitamin C in immortalized mouse embryonic fibroblasts

Down-regulated for at least 1.5 folds

**Enrichment of genes in Extracellular matrix functional category fold p**

a disintegrin-like and metallopeptidase with thrombospondin-like, 1 4.8 0.004

a disintegrin-like and metallopeptidase with thrombospondin-like, 4 2.4 0.002

a disintegrin-like and metallopeptidase with thrombospondin type 1 motif, 1 1.7 0.001

a disintegrin-like and metallopeptidase with thrombospondin type 1 motif, 10 1.5 0.008

a disintegrin-like and metallopeptidase with thrombospondin type 1 motif, 15 7.8 0.0004

a disintegrin-like and metallopeptidase with thrombospondin type 1 motif, 2 2.5 0.0002

a disintegrin-like and metallopeptidase with thrombospondin type 1 motif, 4 1.7 0.006

a disintegrin-like and metallopeptidase with thrombospondin type 1 motif, 9 2.6 0.008

asporin 4.9 0.0001

bone morphogenetic protein 4 2.1 0.0001

C-type lectin domain family 3, member b 8.4 0.0008

cell adhesion molecule with homology to L1CAM 2.2 0.004

collagen, type I, alpha 1 1.7 0.003

collagen, type II, alpha 1 5.0 0.0003

collagen, type III, alpha 1 3.1 0.002

collagen, type V, alpha 3 1.8 0.002

collagen, type VI, alpha 1 2.6 0.002

collagen, type VI, alpha 2 3.7 0.003

collagen, type VIII, alpha 2 4.5 0.001

collagen, type XI, alpha 1 3.7 0.0001

collagen, type XII, alpha 1 1.7 0.005

collagen, type XIV, alpha 1 13.2 0.002

collagen, type XXVII, alpha 1 1.7 0.006

dermatopontin 3.1 0.001

ectonucleoside triphosphate diphosphohydrolase 2 3.7 0.0008

EGF-like-domain, multiple 6 18.3 0.0001

extracellular matrix protein 2, female organ and adipocyte specific 4.4 0.002

fibrillin 1 1.8 0.004

fibrillin 2 4.4 0.0002

fibromodulin 2.4 0.001

glypican 3 1.9 0.003

glypican 4 1.6 0.0009

hemicentin 1 2.6 0.008

Kazal-type serine peptidase inhibitor domain 1 2.1 0.001

keratocan 10.6 0.0004

laminin, alpha 4 2.5 0.003

latent transforming growth factor beta binding protein 4 1.6 0.005

lumican 3.4 1E-05

matrilin 2 1.6 0.0008

matrix metallopeptidase 17 1.8 0.0005

microfibrillar-associated protein 1B

microfibrillar-associated protein 2 1.9 0.001

microfibrillar-associated protein 4 2.7 0.0006

microfibrillar associated protein 5 2.3 0.003

netrin G1 4.2 0.0004

nidogen 2 4.0 0.0003

osteoglycin 4.3 0.002

podocan 5.2 1E-05

proline arginine-rich end leucine-rich repeat 2.6 0.0005

solute carrier family 1 (glial high affinity glutamate transporter), member 3 3.2 0.0002

SPARC related modular calcium binding 2 2.4 0.002

sparc/osteonectin, cwcv and kazal-like domains proteoglycan 3 2.3 0.0007

spondin 2, extracellular matrix protein 2.2 0.0002

tissue inhibitor of metalloproteinase 3 2.0 0.0007

transforming growth factor, beta induced 1.8 0.002

von Willebrand factor A domain containing 1 2.0 0.0001

wingless-related MMTV integration site 16 2.0 0.005

zona pellucida glycoprotein 3 3.0 0.0008

Down-regulated for at least 1.5 folds

**Enrichment of genes in Cell adhesion functional category fold p**

adhesion molecule with Ig like domain 3 1.6 0.002

amine oxidase, copper containing 3 2.6 0.009

amyloid beta (A4) precursor-like protein 1 2.3 0.004

angiotensinogen (serpin peptidase inhibitor, clade A, member 8) 2.8 0.002

BCL2-like 11 (apoptosis facilitator) 2.0 0.002

biregional cell adhesion molecule-related/down-regulated 2.1 0.001

by oncogenes (Cdon) binding protein

cadherin 11 1.5 0.002

cadherin-like 26 2.0 1E-05

calsyntenin 2 3.5 0.0005

carboxypeptidase X 2 (M14 family) 3.8 0.0006

catenin (cadherin associated protein), alpha 2 2.9 0.0007

cell adhesion molecule with homology to L1CAM 2.2 0.004

cell adhesion molecule-related/down-regulated by oncogenes 2.4 0.004

cerebral endothelial cell adhesion molecule 1.9 0.002

chemokine (C-X3-C motif) ligand 1 1.9 0.004

claudin 1 2.2 0.002

collagen, type II, alpha 1 5.5 0.0003

collagen, type V, alpha 3 1.8 0.008

collagen, type VI, alpha 1 2.6 0.002

collagen, type VI, alpha 2 3.7 0.003

collagen, type VIII, alpha 2 4.5 0.001

collagen, type XI, alpha 1 3.7 0.0001

collagen, type XII, alpha 1 1.7 0.005

collagen, type XIV, alpha 1 13.2 0.002

collagen, type XX, alpha 1 1.6 0.008

collagen, type XXVII, alpha 1 1.7 0.006

dermatopontin 3.1 0.001

EGF-like-domain, multiple 6 18.3 0.0001

embryonal Fyn-associated substrate 1.6 0.006

endomucin 1.9 0.002

FAT tumor suppressor homolog 4 3.7 0.001

fibulin 5 3.3 0.003

integrin alpha 11 3.0 0.0007

integrin alpha 8 2.3 0.002

intercellular adhesion molecule 1 2.2 0.005

laminin, alpha 4 3.1 0.008

microfibrillar-associated protein 4 2.7 0.0006

neural precursor cell expressed, developmentally down-regulated gene 9 1.8 0.009

nidogen 2 4.0 0.0003

phosphoglucomutase 5 2.3 0.002 protocadherin beta 14 1.9 0.003

protocadherin beta 15 1.7 0.006

protocadherin beta 16 1.5 0.002

Ras association and DIL domains 1.9 0.002

roundabout homolog 2 8.6 0.002

tumor necrosis factor alpha induced protein 6 1.7 0.002

spondin 2, extracellular matrix protein 2.2 0.0002

sushi, von Willebrand factor type A, EGF and pentraxin domain containing 1 2.1 0.003

thrombospondin 2 1.6 0.004

thyroid hormone receptor alpha; similar to thyroid hormone receptor 1.6 0.002

transforming growth factor, beta 2 2.2 0.0004

transforming growth factor, beta induced 1.8 0.002

Down-regulated for at least 1.5 folds

**Enrichment of genes in Collagen functional category fold p**

C1q and tumor necrosis factor related protein 3 3.2 0.007

C1q and tumor necrosis factor related protein 5 2.1 0.0005

C1q and tumor necrosis factor related protein 7 9.3 0.0002

collagen, type I, alpha 1 1.7 0.003

collagen, type II, alpha 1 5.0 0.0003

collagen, type III, alpha 1 3.1 0.002

collagen, type V, alpha 3 1.8 0.008

collagen, type VI, alpha 1 2.6 0.002

collagen, type VI, alpha 2 3.7 0.003

collagen, type VIII, alpha 2 4.5 0.001

collagen, type XI, alpha 1 3.7 0.0001

collagen, type XII, alpha 1 1.7 0.005

collagen, type XIV, alpha 1 13.2 0.002

collagen, type XX, alpha 1 1.6 0.008

collagen, type XXIII, alpha 1 2.6 0.003

collagen, type XXVII, alpha 1 1.7 0.006

collectin sub-family member 12 1.7 0.008

procollagen-proline, 2-oxoglutarate 4-dioxygenase, alpha II polypeptide 1.7 0.002

scavenger receptor class A, member 3 2.8 0.0001

Down-regulated for at least 3 folds

**Enrichment of genes in Extracellular matrix functional category fold p**

a disintegrin-like and metallopeptidase with thrombospondin-like, 1 4.8 0.004

a disintegrin-like and metallopeptidase with thrombospondin type 1 motif, 15 7.8 0.0004

C-type lectin domain family 3, member b 8.4 0.0008

collagen, type II, alpha 1 5.0 0.0003

collagen, type III, alpha 1 3.1 0.002

collagen, type VIII, alpha 2 4.5 0.001

collagen, type XI, alpha 1 3.7 0.0001

collagen, type XIV, alpha 1 13.2 0.002

dermatopontin 3.1 0.001

ectonucleoside triphosphate diphosphohydrolase 2 3.7 0.0008

EGF-like-domain, multiple 6 18.3 0.0001

extracellular matrix protein 2, female organ and adipocyte specific 4.4 0.002

fibrillin 2 4.4 0.0002

keratocan 10.6 0.0004

laminin, alpha 4 3.1 0.008

lumican 3.4 1E-05

netrin G1 4.2 0.0004

nidogen 2 4.0 0.0003

osteoglycin 4.3 0.002

podocan 5.2 1E-05

solute carrier family 1 (glial high affinity glutamate transporter), member 3 3.2 0.0002
